# Conditional GWAS of non-CG transposon methylation in *Arabidopsis thaliana* reveals major polymorphisms in five genes

**DOI:** 10.1371/journal.pgen.1010345

**Published:** 2022-09-09

**Authors:** Eriko Sasaki, Joanna Gunis, Ilka Reichardt-Gomez, Viktoria Nizhynska, Magnus Nordborg

**Affiliations:** 1 Gregor Mendel Institute of Molecular Plant Biology, Austrian Academy of Sciences, Vienna BioCenter (VBC), Vienna, Austria; 2 Faculty of Science, Kyushu University, Fukuoka, Japan; 3 Max Planck Institute of Molecular Cell Biology and Genetics, Dresden, Germany; University of Minnesota, UNITED STATES

## Abstract

Genome-wide association studies (GWAS) have revealed that the striking natural variation for DNA CHH-methylation (mCHH; H is A, T, or C) of transposons has oligogenic architecture involving major alleles at a handful of known methylation regulators. Here we use a conditional GWAS approach to show that CHG-methylation (mCHG) has a similar genetic architecture—once mCHH is statistically controlled for. We identify five key *trans*-regulators that appear to modulate mCHG levels, and show that they interact with a previously identified modifier of mCHH in regulating natural transposon mobilization.

## Author summary

DNA methylation is an epigenetic mark common across eukaryotes. It is associated with transcriptional silencing, in particular of transposable elements. Multiple elements, including epigenetic inheritance, shape DNA methylation patterns, and the complexity makes it challenging to dissect the regulation. Our work on the 1001 Arabidopsis Epigenomes project led to the unexpected discovery that much of the natural variation for CHH methylation (mCHH; H is A, T, or C) on transposable elements could be attributed to allelic variation in three genes known to be involved in epigenetic regulation. However, our analysis of methylation in other sequence contexts (mCHG and mCG) revealed no genetic regulator.

Here we show that if mCHG variation is analyzed while taking mCHH into account, mCHG is also strongly influenced by allelic variation in a small number of genes with known or highly plausible direct roles in epigenetic regulation. The presence of common allelic variation of large effect is suggestive of some form of local adaptation. The nature of this adaptation remains obscure, but we present further evidence that allelic variation regulating DNA methylation influences transposon mobilization.

## Introduction

Organisms have developed defense systems to protect the genome from transposable elements, which can act as ‘selfish genes’ and cause considerable damage [1,2]. DNA methylation is a major component of genome defense that is found in both mammals and plants, albeit with significant differences [2,3]. For instance, whereas mammals mostly have CG methylation (mCG), methylation in plants also occurs in the CHG and CHH contexts (H is A, T, or C). mCG in plants is known to be maintained through both mitosis and meiosis by *DNA METHYLTRANSFERASE 1* (*MET1; DNMT1* in humans)—in contrast to CHH methylation (mCHH), which is re-established after cell division by several pathways, including the RNA-directed DNA methylation (RdDM) pathway and the *CHROMOMETHYLASE 2* (*CMT2*) pathway [4,5]. Unlike mCG, which is stably inherited, mCHH behaves like a molecular phenotype and is strongly influenced by the environment, such as growth temperature [6] and stress [7]. CHG methylation (mCHG) falls somewhere between mCG and mCHH in the sense that it can be maintained via positive feedback between *CHROMOMETHYLASE3* (*CMT3*) and *KRYPTONITE* (*KYP*), which recognize dimethylation of histone 3 lysine 9 (H3K9me2) and mCHG, respectively [8–11]. Molecular mechanisms aside, the forces shaping variation in DNA methylation remain obscure, and the same is true for its biological significance [7,12–19]. Previously, we demonstrated the existence of large-scale geographic clines for DNA methylation in *A*. *thaliana*, and used genome-wide association studies (GWAS) to show that mCHH on transposons is heavily influenced by major polymorphisms at *trans*-acting modifiers corresponding to known DNA methylation regulators in silencing pathways: *CMT2*, *NUCLEAR RNA POLYMERASE D1B* (*NRPE1*), *ARGONAUTE 1* (*AGO1*), and *AGO9* [6,20]. In particular, an allele of *NRPE1* (named *NRPE1’*) strongly affects mCHH levels in RdDM-targeted transposons. *NRPE1*, which is the largest subunit of RNA polymerase V, localizes to promoter regions of relatively young transposons [21], and Baduel et al. [22] recently showed that the *NRPE1’* allele is associated with recent transposon mobilization, presumably through its effect on DNA methylation. All in all, these findings point to a highly variable genome defense system.

While both mCHG and mCG showed high heritability, GWAS yielded little in terms of significant associations. This might be because these “traits” are highly polygenic, or because they are at least partly transgenerationally inherited, and hence do not behave like standard phenotypes (i.e. the observed trait does not necessarily reflect current genetics or environment). In this study, we revisit mCHG variation and use a conditional GWAS approach to reveal that multiple major alleles at *trans*-acting regulators modify this type of methylation, and are also associated with transposon mobilization.

## Results

### mCHG is strongly correlated with mCHH

**Fig 1:**
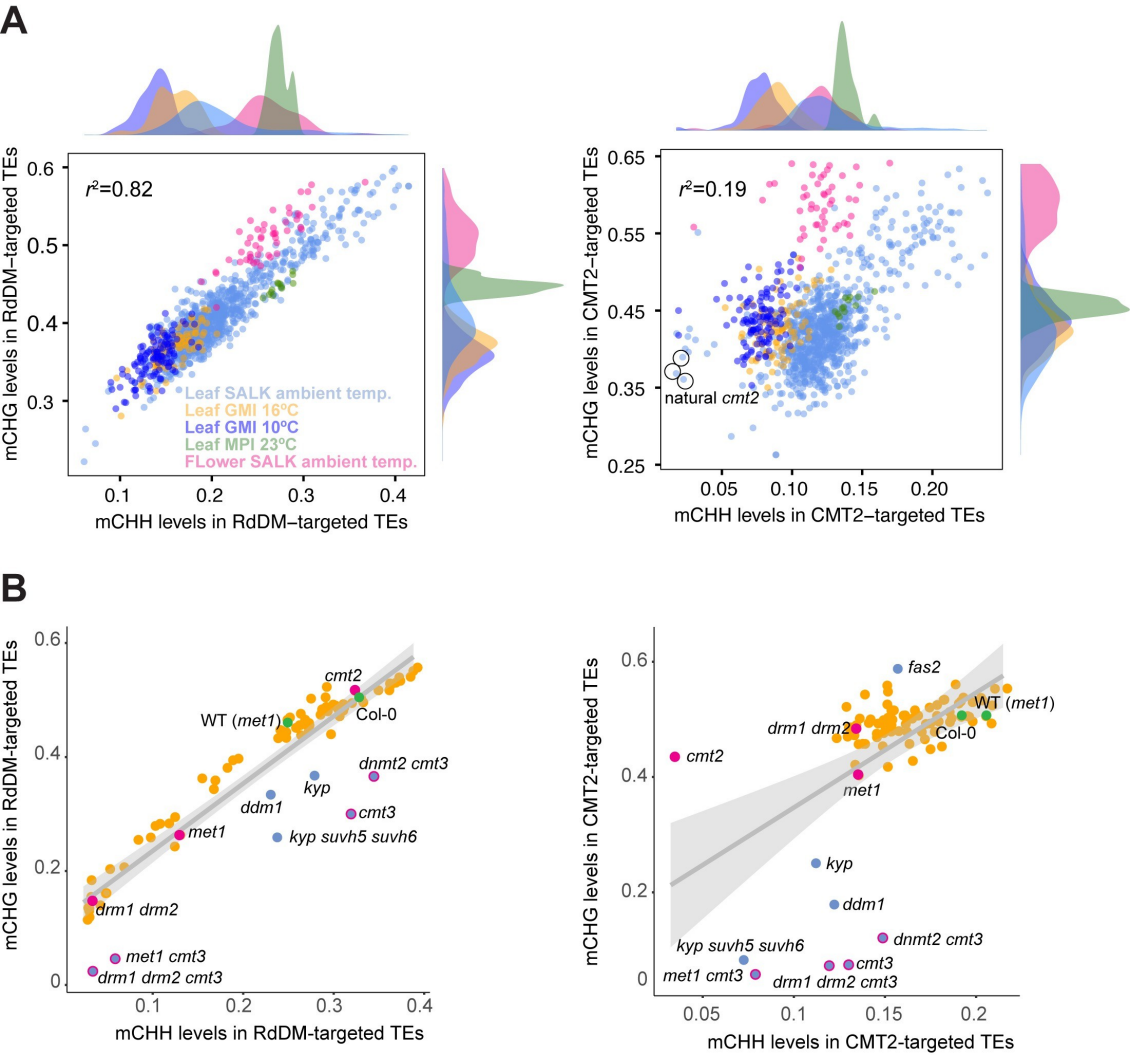
Covariance between mCHH and mCHG levels across individuals. **(A)** Correlation of genome-wide average mCHH and mCHG levels in RdDM- and CMT2-targeted transposons across 1028 natural inbred lines measured in different conditions (including 79 lines measured in more than one condition; see [20]) Colors correspond to environments/tissues. Plots on axes show marginal densities. Circled lines carry known natural null alleles of *CMT2*. **(B)** The same correlation as in A, but for 84 epigenetics-related loss-of-function mutants [27]. Gray areas indicate 99% confidence intervals around the linear regression lines. Green points denote “wildtypes” (see [27]); magenta, major DNA methyltransferases. Blue points denote lines with a highly significant effect on mCHG.

Our starting point is the observation that mCHG and mCHH levels on transposons are strongly correlated in the 1001 Epigenomes data set [20], especially for RdDM-targeted transposons (Fig 1A; see Methods). Much of the variation in these two traits is due to differences in the environment—including tissue, which can be viewed as a cellular environment. It is important to note that these are not single-cell data, and that differences in DNA methylation levels may arise simply because of different levels of cell division states between samples [23], although hyper-mCHG in flower tissue samples probably also reflect tissue-specific *CMT3* expression [24–26]. At the same time, variation across individuals is enormous even when controlling for known tissue and environmental effects, as observed in the largest leaf sample data set (“Leaf SALK ambient temperature”; *n* = 846).

Interestingly, the covariance between mCHH and mCHG showed the same pattern in data generated by knocking out known or potential DNA methylation regulators in the same genetic background (Fig 1B) [27]. This demonstrates strong co-regulation of these types of methylation, in particular for RdDM-targeted transposons. Loss of RdDM regulation, such as in the double mutant of *DOMAINS REARRANGED METHYLASE (DRM) drm1 drm2*, causes loss of almost all mCHH and mCHG because mCHG in these regions is mainly established via siRNA [28]. At the same time, the data demonstrate that some genetic perturbations can affect one type of methylation much more than the other. For example, knocking out mCHG-regulators, like *CMT3* or *KYP*, affects mCHG levels much more than mCHH levels.

Based on these observations, it is clear that a substantial fraction of variation in non-CG methylation is due to factors that affect both mCHH and mCHG. Without replicate measurements, it is difficult to say what fraction of these factors is genetic and what is environmental, but, irrespective of this, we hypothesized that the substantial covariance could reduce the power of GWAS for either mCHH or mCHG (when using a standard univariate model), and that an analysis accounting for this covariance might perform better [29–31]. In essence, we sought to simplify a complex trait by breaking it into constituent parts [32–34]. This worked, as we shall see below.

### Conditional GWAS reveals new associations

Multi-trait GWAS can be carried out using transformations (e.g., the difference or ratio between traits), conditional approaches in which one or more traits are accounted for as covariates, or full multivariate models [29–31]. We tried several different approaches, including a multivariate GWAS, but this paper will focus on results from a conditional GWAS of mCHG with mCHH as a covariate (denoted mCHG_|mCHH_) because this gave the clearest results. Other approaches produced consistent results, and we will briefly discuss them later.

**Fig 2:**
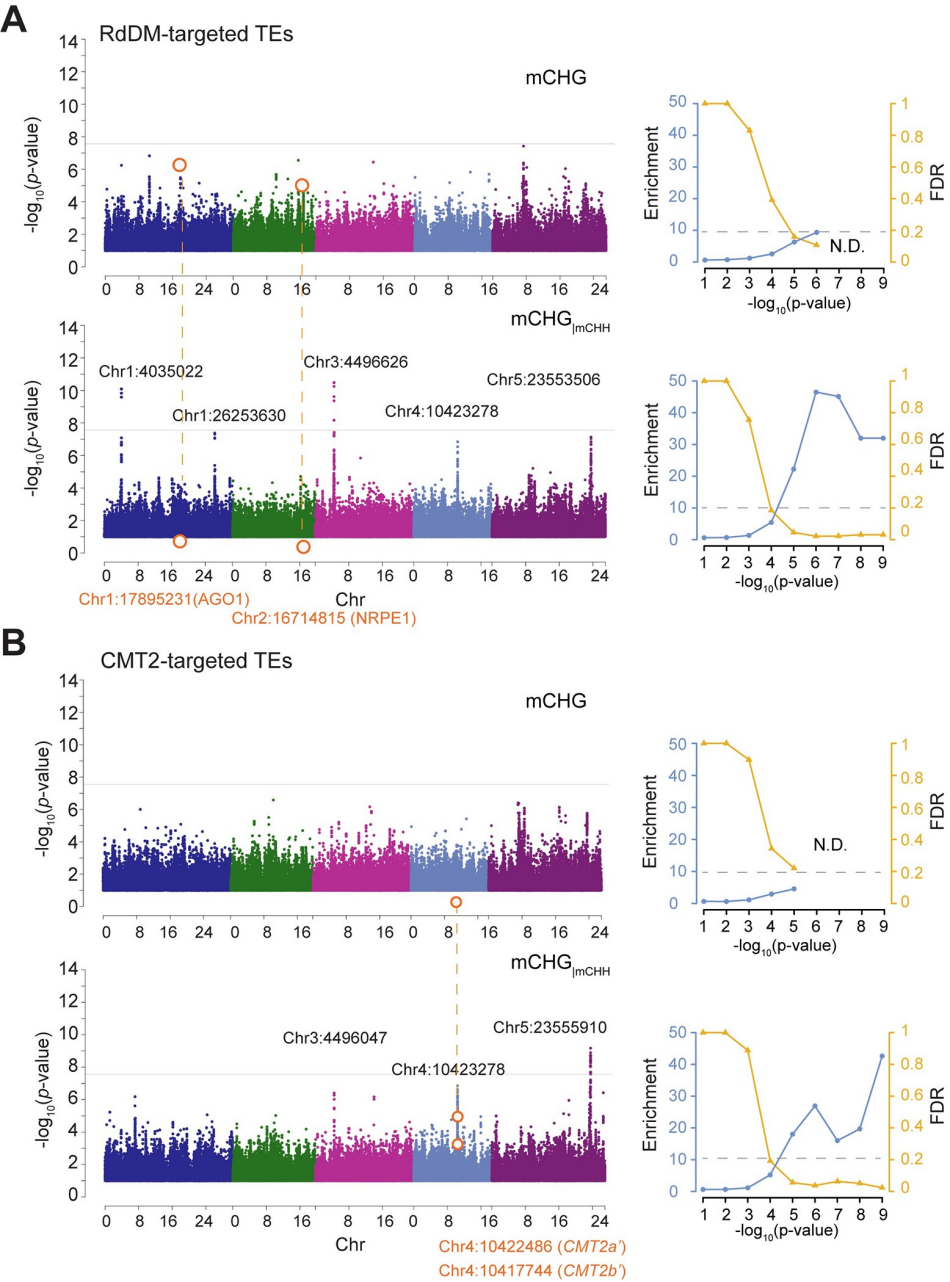
Comparison of univariate and conditional GWAS of mCHG. The analysis was done separately for (**A)** RdDM-targeted and (**B**) CMT2-targeted transposons, using the 774 lines in the global panel from the 1001 Epigenomes Project (“SALK leaf in ambient temperature”; see Fig 1). For each case, the upper Manhattan plot shows univariate GWAS of mCHG and the lower GWAS of mCHG controlling for mCHH. Horizontal gray lines show genome-wide significance (*p* = 0.05 after Bonferroni-correction). The line plots show enrichment of *a priori* genes and FDR (see text), with horizontal dashed lines indicating an FDR of 20%.

Fig 2 contrasts the performance of univariate and conditional GWAS. As previously noted, univariate GWAS of mCHG does not yield any significant associations despite moderate SNP-heritability (37% and 38% for RdDM- and CMT2-targeted transposons, respectively). As expected given the very strong correlation between mCHH and mCHG on RdDM-targeted transposons, the strong associations at *AGO1* and *NRPE1* found in univariate GWAS of mCHH [20] give rise to associations for mCHG as well, but they are not genome-wide significant (*AGO1* Chr1:17895231, -log_10_*p*-value = 6.51 and *NRPE1* Chr2:16714815, -log_10_*p*-value = 5.23). The previously identified *CMT2*-association with mCHH on CMT2-targeted transposons [6,20,35,36] is not apparent, consistent with the much weaker correlation between mCHH and mCHG on these transposons.

The contrast between these univariate GWAS results and the conditional analysis is striking. GWAS for mCHG while controlling for mCHH (mCHG_|mCHH_) revealed five clear peaks for RdDM-targeted transposons, three of which were also found for CMT2-targeted transposons. The peaks are above or near genome-wide significance using a conservative threshold (Figs 2 and S1 and S1 Table). As will be discussed further below, these associations jointly explain 30.5% and 14.8% of mCHG_|mCHH_ in RdDM- and CMT2-targeted transposons, respectively. The previously mentioned *AGO1* and *NRPE1* associations disappear completely, consistent with their being strongly correlated with mCHH, and hence controlled for. The improved performance of conditional GWAS is also evident from a massive enrichment of associations near *a priori* candidates (see Methods and [37]). With conditional GWAS, we observed an up to 45-fold excess of associations near annotated epigenetic modifiers [20,35], compared to an 4- to 10-fold excess for the univariate analyses (Fig 2).

The enrichment for *a priori* candidates also allows us to estimate a False Discovery Rate (FDR) for this set of genes [37]. Four of the five major associations can be identified with *a priori* candidates at very low FDRs (e.g. FDR < 0.05 using a significance threshold of -log_10_*p*-value > 6; see Fig 2), but it is notable that FDR is low even for associations that are nowhere near genome-wide significance. This is not true for the univariate analyses. For example, at an FDR of 20%, univariate GWAS of mCHG identifies only *AGO1* and *NRPE1* for RdDM-targeted transposons, and nothing for CMT2-targeted transposons (S2 Table), whereas conditional GWAS of mCHG_|mCHH_ generates a long list of known regulators of mCHG (S2 Table). For RdDM-targeted transposons, we find four *a priori* genes above or near genome-wide significance: the previously mentioned *CMT2* and *CMT3*, plus *MIR823a*, which encodes a microRNA targeting *CMT3* [26,38], and *MULTICOPY SUPPRESSOR OF IRA1* (*MSI1*), likely a component of chromatin assembly factor (CAF-1) responsible for proper heterochromatin formation with *FASCIATA1* (*FAS1*) and *FASCIATA2* (*FAS2*) [39,40]. At lower significance levels, we find *REPRESSOR OF SILENCING 3* (*ROS3*) and *DNA METHYLTRANSFERASE 2* (*DNMT2*). *ROS3* is a DNA demethylase that interacts with *REPRESSOR OF SILENCING 1* (*ROS1*) [41] and *DNMT2* is associated with histone deacetylation [42] and RNA methyltransferase activity [43] (and could therefore be a false positive since the effects on DNA methylation is under debate; see [42,43]). For CMT2-targeted transposons, the list of associated *a priori* candidates includes four genes also associated with RdDM-targeted transposons, namely *MIR823A*, *CMT2*, *ROS3*, and *MSI1*, but there are also two specific genes, *FAS1* and *DECREASED DNA METHYLATION 1* (*DDM1*), a chromatin-remodeler responsible for heterochromatin formation [44,45]. As shown in Fig 1B, *ddm1* strongly reduces mCHG, and *fas2*, a functionally redundant *FAS1* homolog, increases CMT2-targeted mCHG in an mCHH-independent manner [27].

### Genes underlying major associations

#### On searching for causal genes

**Fig 3:**
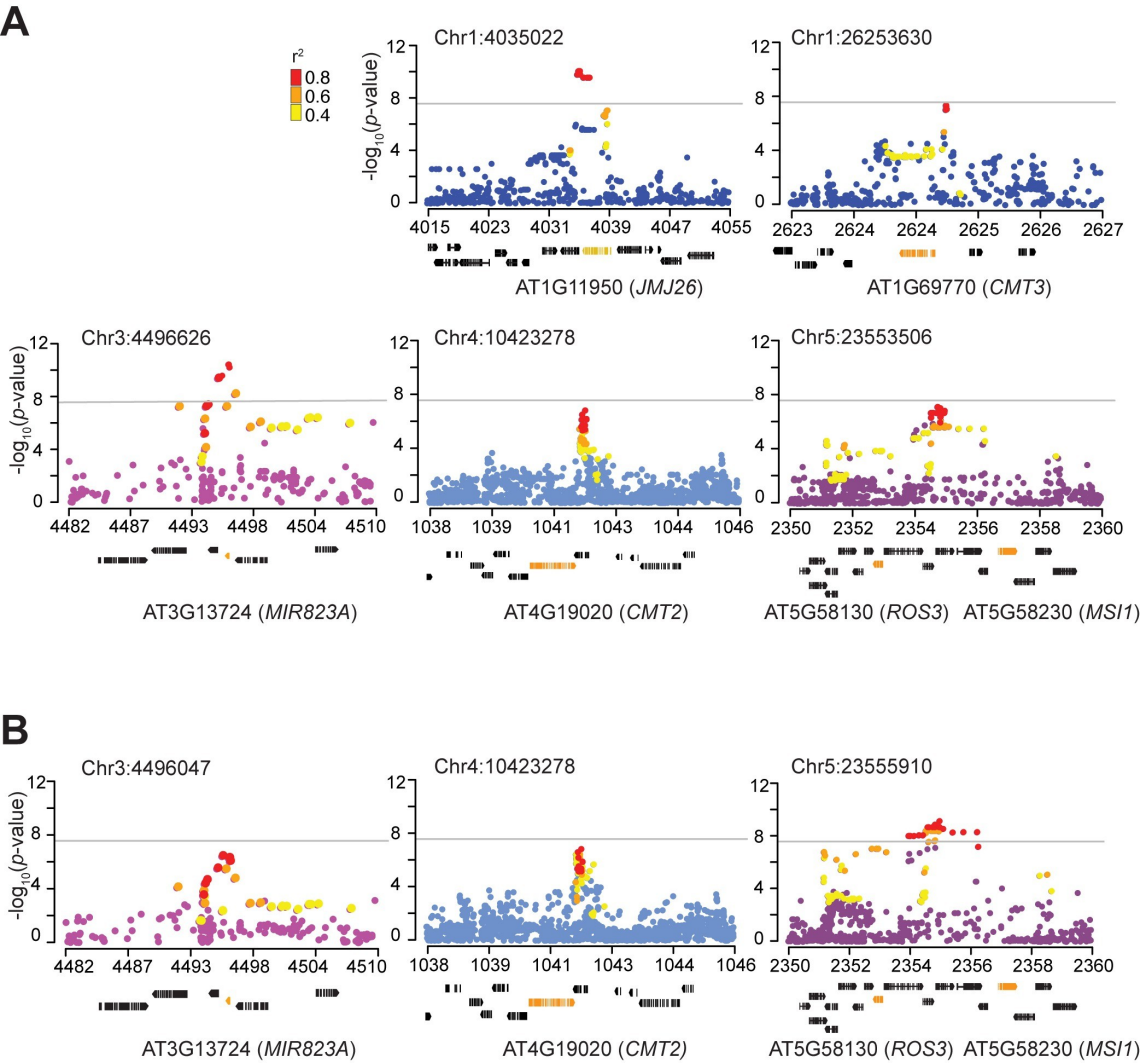
Candidate loci underlying mCHG_|mCHH_ variation. Zoomed-in Manhattan plots around conditional GWAS peaks in Fig 2 for **(A)** RdDM- and **(B)** CMT2-targeted transposons. Gene models below the plots show the candidate genes in orange (see Table 1). Colored dots represent SNPs in strong linkage disequilibrium with top SNPs.

Identifying causal genes and mechanisms from GWAS results is notoriously difficult [46]. Peaks of association often cover multiple genes—this is certainly true in the gene-dense genome of *A*. *thaliana* [37]—and functional annotation is of limited use for complex traits with unknown genetic basis. We are in a stronger position, however, because, just like in our previous work on mCHH [35], the significant GWAS peaks (Fig 2) are narrow, and clearly pinpoint genes *a priori* known to be specifically involved in well-defined molecular phenotypes (Table 1 and Fig 3). Hence we will discuss these candidates, and the indirect evidence supporting their causal role.

**Table 1:** Significant conditional GWAS hits for mCHG_|mCHH_*.

SNPs	-log_10_ (*p*-value)	MAC	Distance from *a priori* gene (bp)	Genes within 15K bp*
*mCHG*_*|mCHH*_ *in RdDM-targeted transposons*				
Chr1:4035022	10.1	77	NA	AT1G11930, AT1G11940, AT1G11950 (*JMJ26*), AT1G11960
Chr1:26253630	7.38	290	50	**AT1G69770 (*CMT3*)**
Chr3:4496626	10.48	207	196	AT3G13700, AT3G13710 (*PRA1*.*F4*), AT3G13720 (*PRA1*.*F3*), **AT3G13724 (*MIR823A*)**, AT3G13730 (*SYP90D1*)
Chr4:10423278	6.85	301	2069	**AT4G19020 (*CMT2*)**, AT4G19030 (*NLM1*), AT4G19035 (*LCR7*)
Chr5:23553506	7.14	99	2506	AT5G58200 (*ECT10*), AT5G58200, AT5G58210, AT5G58220 (*TTL*), **AT5G58230 (*MSI1*)**, AT5G58240 (*FHIT*)
*mCHG*_*|mCHH*_ *in CMT2-targeted transposons*				
Chr3:4496047	6.42	210	775	AT3G13700, AT3G13710 (*PRA1*.*F4*), AT3G13720 (*PRA1*.*F3*), **AT3G13724 (*MIR823A*)**, AT3G13730 (*SYP90D1*)
Chr4:10423278	6.86	301	2069	**AT4G19020 (*CMT2*)**, AT4G19030 (*NLM1*), AT4G19035 (*LCR7*)
Chr5:23555910	9.17	64	102	AT5G58200, AT5G58210, AT5G58220 (*TTL*), **AT5G58230 (*MSI1*)**, AT5G58240 (*FHIT*), AT5G58250, AT5G58260 (*NdhN*)

* Bold denotes *a priori* epigenetic regulators

#### Multi-layered direct *CMT3* regulation affects mCHG variation

**Fig 4:**
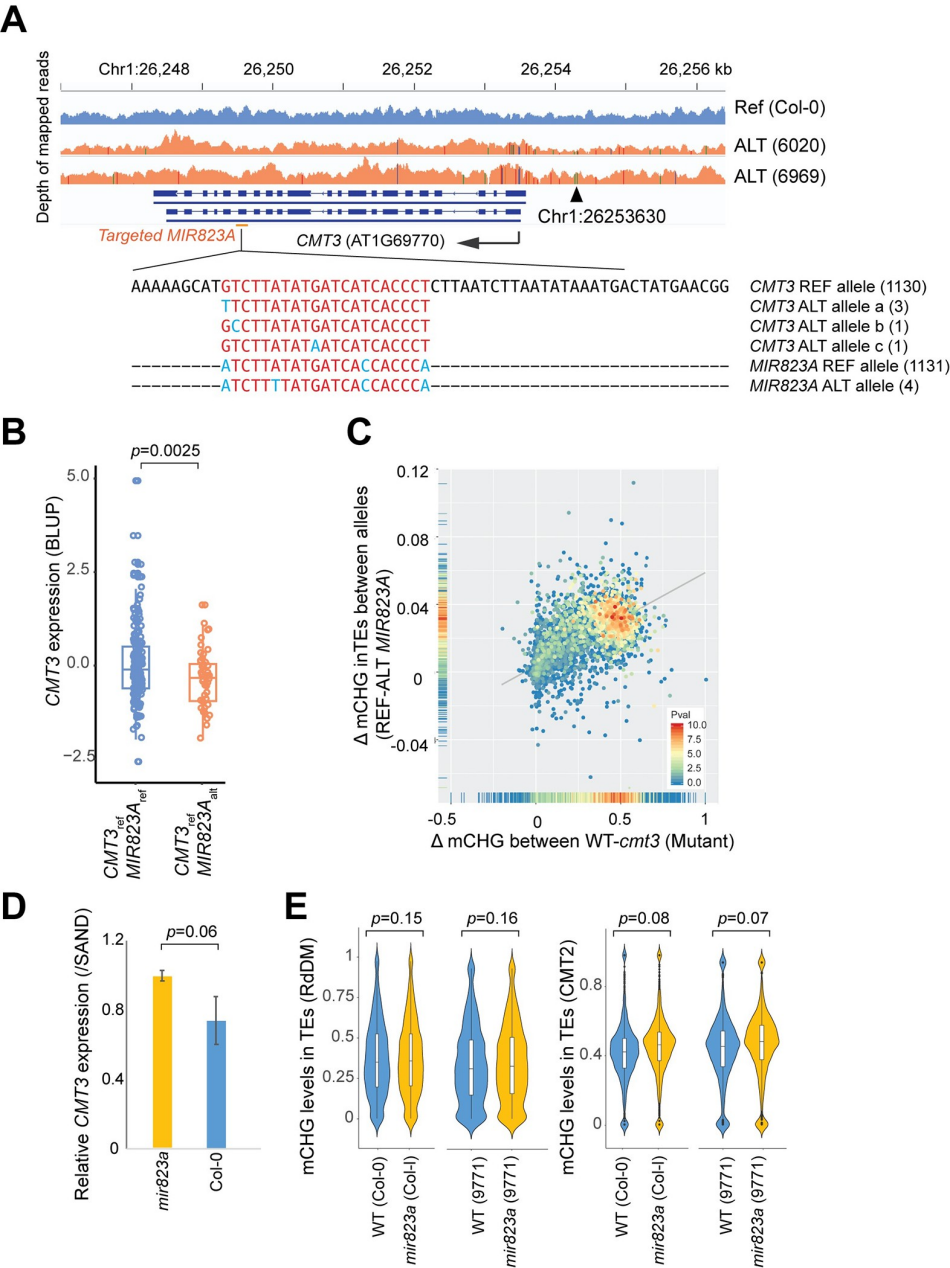
Effects of *MIR823A* on mCHG levels. **(A)** Polymorphisms in the miRNA region of *MIR823A* and the target in *CMT3* in 1135 natural lines. Differences from the *CMT3* reference sequence are shown in light blue. Haplotype counts are shown in parentheses. **(B)** Estimated effects of *MIR823A* alleles on *CMT3* expression in natural lines with individual values (Welch’s t-test, two-tailed, *CMT3* expression was collected from transcriptome data published in [20]). **(C)** Scatter plot comparing the average allelic effect of the *MIR823A* polymorphism with the effect of *cmt3* in the Col-0 background. Dots represent individual transposons, and the colors show the significance of the allelic effects as -log_10_p-value in GWAS. **(D)** *CMT3* expression in early embryos with standard errors in WT (Col-0) and *mir823a*. Significance of the difference in mean tested using Welch’s t-test (*n* = 3, one-tailed). **(E)** The distribution of mCHG levels in RdDM and CMT2-targeted transposons, comparing knock-outs of *MIR823A* to wild-type in different backgrounds.

Based on mutant phenotypes, *CMT3* is a strong *a priori* candidate for regulating mCHG (Fig 1). Consistent with this, one of the five major associations from GWAS of mCHG_|mCHH_ pinpoints *CMT3* and another *MIR823a*, a gene encoding a microRNA that directly down-regulates *CMT3* by cleaving its transcripts during early embryogenesis [26]. The most significant peak for mCHG_|mCHH_ on RdDM-targeted transposons was located 196 bp downstream of *MIR823a*, while the peak corresponding to *CMT3* was located 50 bp upstream of the gene in a potential regulatory region (Figs 4A and S4A and Table 1). GWAS for CMT2-targeted transposons also pinpointed *MIR823a*, although the association is weaker, and the most significant SNP is further away (Fig 3B and Table 1). This association was also recently found by Hüther et al. [47] using GWAS for unconditional mCHG levels of individual transposons.

The *MIR823A* polymorphism appears to almost exclusively affect mCHG (S2 and S3 Figs), primarily targeting the same transposons as a *CMT3* knock-out, as expected if the former directly regulates the latter (Fig 4C). Consistent with this interpretation, lines carrying the non-reference *MIR823A* allele and a *CMT3* reference allele showed lower *CMT3* expression as well as lower mCHG (Fig 4B). The specificity of the phenotypic effects and known regulatory mechanism provides strong evidence for a direct causal role for these genes.

Knock-outs of *MIR823A* in several backgrounds affected *CMT3* expression and mCHG in a manner consistent with this regulatory model [26], although the effects on methylation were very weak (Figs 4D, 4E and S4D). The natural polymorphism almost certainly does not involve a loss of function, as there is no common polymorphism in the 21-nt mature miRNA region of *MIR823A*, nor in the target region of *CMT3* (Fig 4A). There are also no other non-synonymous polymorphisms in *CMT3* significantly associated with the different alleles. There is no SNP in the stem-loop region of *MIR823A* that is significantly associated with mCHG_|mCHH_, and the most significant SNP (Chr3:4496626) does not appear to affect predicted RNA secondary structure [48] (S4C Fig).

#### Further evidence for allelic heterogeneity at *CMT2*

In addition to *de novo* establishment of methylation in heterochromatic regions, *CMT2* plays a role in mCHG maintenance through regulation of H3K9 methylation via a self-reinforcing loop [11,49]. Previous work has identified two common natural alleles, *CMT2a’* and *CMT2b’*, affecting mCHH [6,20,35], but neither appears to affect mCHG (S2, S3 and S5 Figs). Here we identify a new association, 2 kb downstream of *CMT2* (Fig 3), that does appear to affect mCHG (on both RdDM- and CMT2-targeted transposons) (S5 Fig). The top SNP is only weakly correlated with *CMT2a’* and *CMT2b’* (*r*^*2*^_*CMT2b*’_ = 0.37; *r*^*2*^_*CMT2a’*_ = 0.24), but caution is needed when interpreting associations in regions that apparently harbor multiple causal polymorphisms [50].

#### A complex association on chromosome 5 includes two *a priori* genes

The major peak on chromosome 5 was associated with mCHG_|mCHH_ on both RdDM- and CMT2-targeted transposons, although the shape of the peak differs slightly. The strongest association was found for CMT2-targeted transposons and is located 102 bp upstream of a known epigenetic regulator, *MSI1*, but substantial linkage disequilibrium extends over a 30 kb region which also includes another *a priori* gene, *ROS3* (Figs 3 and S6A).

The loss-of-function mutant *ros3* does not show altered mCHG in leaves (Fig 1B)[27]. Loss of *MSI1* causes embryonic lethality [51], but the heterozygous mutant *msi1-2* does not show a significant effect on mCHG levels in leaves either. However, *MSI1* has been copurified with a tesmin/TSO1-like CXC domain-containing protein, a component of a DREAM complex that controls mCHG via repression of *CMT3*, *KYP*, *MET1*, and *DDM1* [52]. In addition, a loss of *FAS2*, a component of CAF-1, which also includes *MSI1*, induces mCHG hypermethylation in CMT2-targeted TEs (Fig 1B right) [27,53]. Therefore, *MSI1* remains a strong candidate. Furthermore, the strongly associated SNP at Chr5:23555910 could explain almost all phenotypic variation associated with the chromosome 5 peak (S6B Fig), making *MSI1* the top candidate, although further experiments are clearly needed. The allelic variation is presumably regulatory, since the SNP is not correlated with any non-synonymous SNPs.

#### A jmjC gene is a novel modifier of mCHG in RdDM-targeted transposons

**Fig 5:**
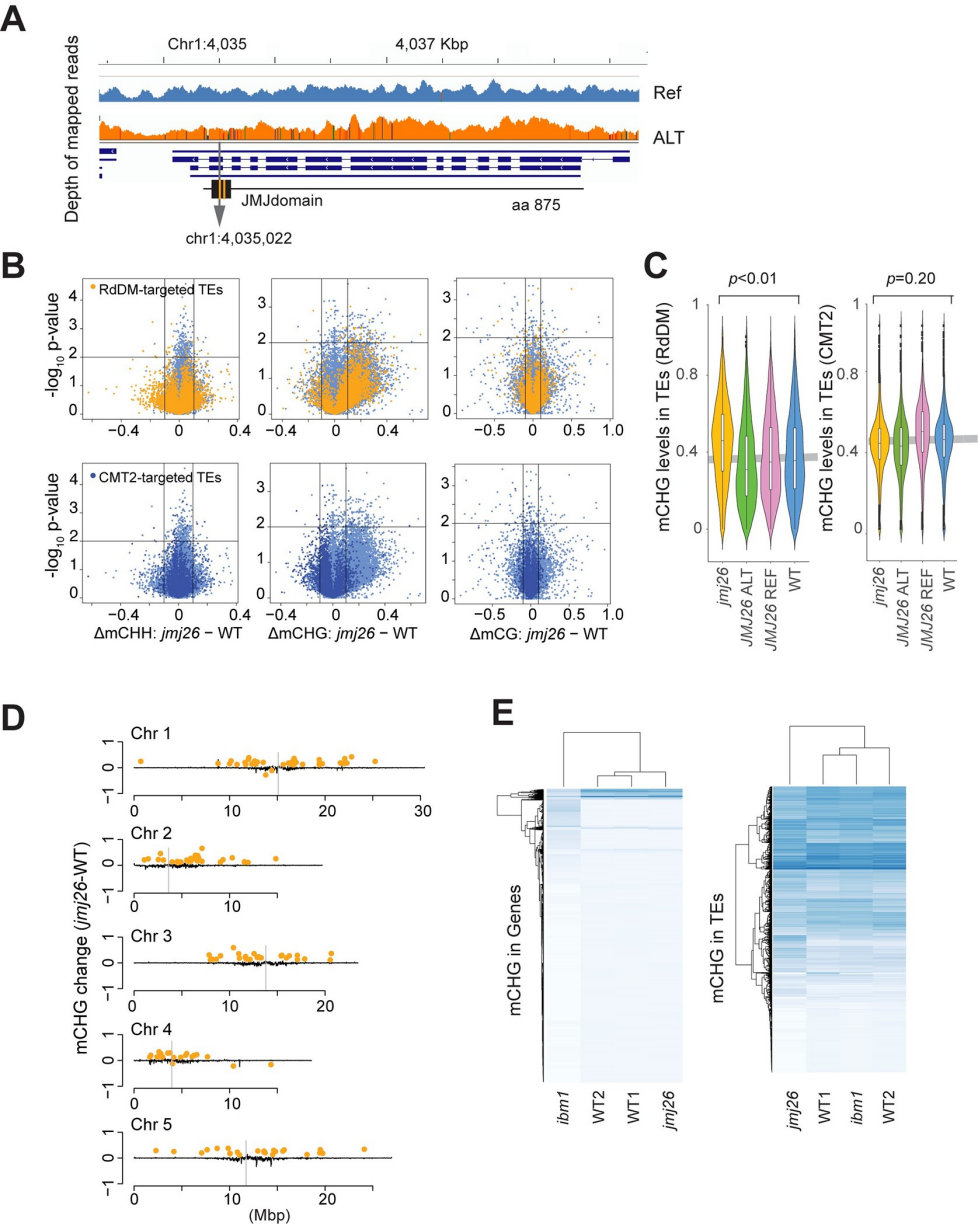
Effects of *JMJ26* on mCHG levels. **(A)** Read density and SNP across the gene model. **(B)** Volcano plots showing the effects on DNA methylation levels of *jmj26* on RdDM- and CMT2-targeted transposons. **(C)** Violin plots showing the distribution of mCHG levels of RdDM- and CMT2-targeted transposons in WT (Col-0), *jmj26*, and two lines with *JMJ26*_ref_ or *JMJ26*_alt_ transgenes in Col-0 *jmj26* background. The gray horizontal line shows the mean value of WT. **(D)** The genome-wide distribution of transposons affected by *JMJ26*. Black lines show differential mCHG levels of *jmj26* from Col-0 in 30 kbp sliding windows. Orange dots show transposons with significantly changed mCHG levels (*n* = 2, *p*<0.01 by two-tailed Welch’s *t*-test & differential mCHG > 0.1 or < -0.1). **(E)** Heat map comparing *JMJ26* and *IBM1* targets. Rows correspond to standardized mCHG levels in each gene or transposon for *jmj26*, *ibm1*, and WTs.

The final peak (Chr1:4035022; -log_10_*p-*value = 10.1) in our study did not pinpoint any *a priori* gene. However, it is highly significant and narrowly centered on the coding region of a jmjC gene, *JUMONJI26* (*JMJ26*) [54]. *JMJ26* is a close homolog of *JMJ25*, also known as *INCREASE IN BONSAI METHYLATION 1* (*IBM1*), a histone H3K9m demethylase targeting genic regions in a *KYP/SUVH4-* and *CMT3*-dependent manner [55,56]. In contrast to *IBM1*, the function of *JMJ26* has barely been studied. Among the most significant associations were two non-synonymous SNPs resulting in amino acid changes (Chr1:4035683, Glu678Val; Chr1:4035690, Leu676Val) in the conserved JMJ domain, suggesting allelic effects on the enzymatic activity (Figs 5 and S7).

To explore the function of *JMJ26*, we measured mCHG levels of a loss-of-function mutant, *jmj26* (Fig 5). Unlike *IBM1* loss-of-function mutants, *jmj26* did not show any morphological phenotype (S8A Fig), but mCHG levels increased significantly in RdDM-targeted transposons (Fig 5B and 5C). Furthermore, *jmj26* showed increased mCHG in pericentromeric transposons, whereas *ibm1*-like hypermethylation was not observed in genic regions (Fig 5D and 5E). Gene expression was barely affected except for a few DNA methylation sensitive genes, including transposon genes (AT5G35935, AT4G01525) (S3 Table). These observations support the functionality of *JMJ26* as a histone demethylase with different targets from *IBM1*, and also make it a strong *a posteriori* candidate for causing the GWAS peak on the left end of chromosome 1.

### Major loci additively affect mCHG and influence transposon activity

**Fig 6:**
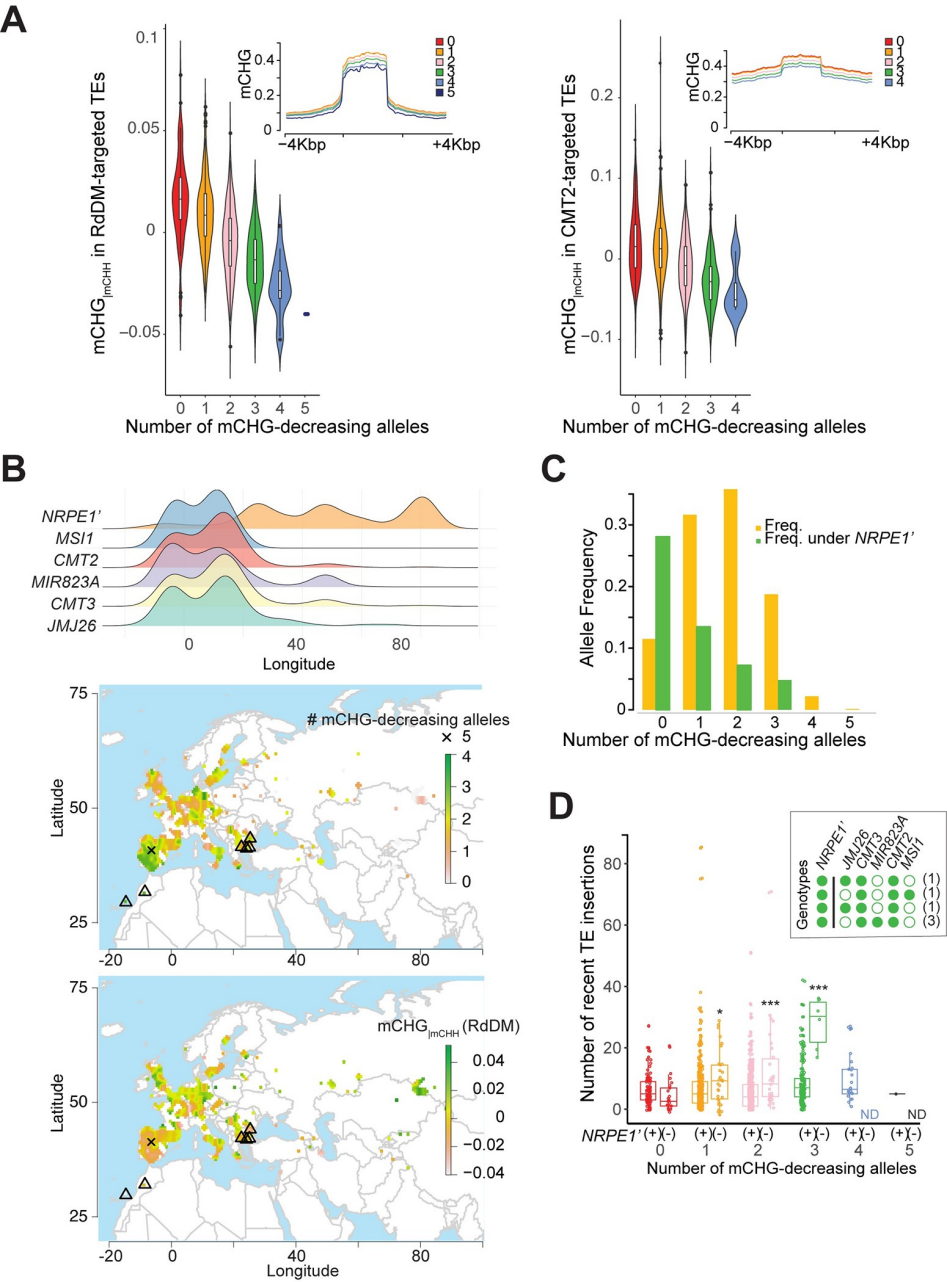
Cumulative effects and geographic distribution of mCHG-decreasing alleles. **(A)** Cumulative effect on mCHG_|mCHH_. Chr1:4035022 (*JMJ26*) was excluded from the analysis of CMT2-targeted transposons as it has no effect. Chr5:2355910 was used for *MSI1*. Inserted plots show mCHG levels around transposons, calculated using 20 sliding windows across the transposon body and flanking regions. **(B)** Longitudinal frequency distribution of mCHG-decreasing and *NRPE1’* alleles (top); geographical distribution of the number of mCHG-decreasing alleles (middle), and mCHG_|mCHH_ levels in RdDM-targeted transposons (bottom). Triangles correspond to lines in the inserted plot of panel D. **(C)** The frequency of distribution of the number of mCHG-decreasing alleles, separately for *NRPE1’* genotypes. **(D)** The number of recent transposon insertions [22] as a function of *NRPE1’* genotype and the number of mCHG-decreasing alleles. Significance was tested using a permutation test (see Methods; *** is *p* < 0.01, * is *p* < 0.1). The insert shows the six observed genotypes with three mCHG-decreasing and the *NRPE1’* non-reference allele. Filled circles indicate mCHH- or mCHG-decreasing alleles. Mapping and statistical testing were performed in R version 3.5.3. Maps were generated using the following packages: maps, ggridges, ggplot, rasrer, tidyverse.

Finally, we investigated how the five major loci jointly shape mCHG variation at the population level. Although some of these genetic variants are clearly interacting at the molecular level (e.g., *MIR823A* directly regulates *CMT3*), the phenotypic effects appear to be additive (Fig 6A), and jointly explain a large fraction of the variation: 30.5% and 14.8% of mCHG_|mCHH_ in RdDM- and CMT2-targeted transposons, respectively, while SNP-based heritabilities of these traits are 65% and 69%. mCHG levels decrease linearly with the number of mCHG-decreasing alleles, and the single line (out of 774) harboring mCHG-decreasing alleles at all five loci exhibits strong hypo-mCHG_|mCHH_ in RdDM-targeted transposons (Fig 6A).

These five loci also affect the geographic distribution of mCHG for both RdDM- and CMT2-targeted transposons. In European lines (*n* = 971), mCHG-decreasing alleles are almost exclusively found in the west, from 0 to 30°E (Figs 6B, S9 and S10). Lines carrying more than three mCHG-decreasing alleles are rare (2.5%), and the one line carrying all five such alleles is from the Iberian peninsula: IP-Mun-0 (9561), an admixed line of relict and western European descent [57].

Interestingly, the longitudinal gradient in the number of mCHG-decreasing alleles is opposite to the longitudinal cline of *NRPE1’* associated with lower mCHH [35] and higher transposon mobilization [22] (Fig 6B). No line was found carrying *NRPE1’* with more than three mCHG-decreasing alleles, and a permutation test shows that this negative correlation is unlikely to be due to population structure (Figs 6C and S10C). A plausible alternative is some form of stabilizing selection on methylation levels: multiple mCHG-decreasing alleles in lines with lower mCHH levels might affect fitness analogously to double knockout mutants of the RdDM and CMT3 pathways that show pleiotropic morphological phenotypes and high transposon transcriptional activity [10,28,49].

To explore this further, we examined the effect of mCHG-negative alleles on transposon mobilization by intersecting our data with those of Baduel et al. [22], who found a significant correlation between recent transposon mobilization and the *NRPE1’* non-reference allele. We found that this effect reflects an epistatic interaction with the five loci reported here: significantly increased transposon mobilization is only found in lines carrying the *NRPE1’* non-reference allele and two or more mCHG-decreasing alleles (*p*-value < 0.01; Figs 6D and S11). In particular, lines carrying three mCHG-decreasing alleles and the *NRPE1*’ non-reference allele have about thirty times more recent transposon insertions than lines carrying no mCHG-decreasing alleles and the *NRPE1’* non-reference allele. Such lines are not closely related and carry different transposon insertions, mostly Copia and MuDR. They are found in two distinct regions: north-western Africa, including the Canary Islands, and the south-eastern Balkans (Fig 6B and S4 Table).

## Discussion

### The genetics of non-CG transposon methylation

Non-CG methylation in plants is associated with gene silencing, especially for transposons. In previous studies, we identified an oligogenic architecture for mCHH on transposons involving major polymorphism at *CMT2*, *NRPE1*, *AGO1*, and *AGO9*—all involved in DNA methylation pathways [6,20,35]. Here we complement this with a conditional GWAS approach and identify five major polymorphisms affecting mCHG, presumably acting via methylation maintenance pathways: *CMT2* (different alleles), *CMT3*, *MIR823A* (directly regulating *CMT3*), *MSI1* (probably), and *JMJ26*. All had previously been shown to affect DNA methylation except *JMJ26*.

We observed major polymorphisms at several of the layers of regulation controlling mCHG, including methylation, de-methylation, and histone modification—even post-transcriptional regulation, in the form of a microRNA targeting the CMT3 pathway directly. The *DDM1* and *FAS1* alleles detected using a non-stringent threshold would further contribute to the fine-tuning of DNA methylation over transposons genome-wide.

The genetic architecture revealed by these studies is very different from the “omnigenic” model [58,59] which has come to represent a typical human GWAS result. It is also different from *trans*-regulation for reported human mCG variation [60,61]. Rather than a diffuse genetic architecture involving huge numbers of loci of small effect and unknown causal relationship to the phenotype, we find a small number of major loci with highly plausible mechanistic connection to the phenotype—and a diffuse background for which we lack the power to dissect further. The results are reminiscent of those found for other clinal traits, like flowering time [37,62], flower color [63], or eye and coat color in mammals [64–66], suggesting that transposon methylation is likewise under selection, most likely as part of a plastic and an environmentally sensitive genome defense system—a notion further supported by the association with transposon mobility reported here and be Baduel et al. [22].

### The power and complexity of conditional GWAS

The performance of GWAS relies on using the right model for the relation between genotype and phenotype. As with other statistical methods, using the wrong model may lead to unpredictable results. However, lack of prior knowledge makes modeling difficult, and univariate linear models are therefore the default in GWAS. Here we focus on correlated traits. Correlations between traits may arise for a variety of reasons, most obviously a shared genetic basis (pleiotropy), and it is clear that such traits should, in principle, be analyzed jointly using some kind of multivariate analysis [29–31,67]. Our results provide a dramatic illustration of the potential benefits of doing so. Despite high heritability, univariate GWAS of mCHG variation failed to detect any significant associations, leading us to conclude that the trait was simply too polygenic [20]. In contrast, GWAS of the same data using a conditional model that controlled for mCHH revealed an oligogenic architecture with five major loci—qualitatively similar to what we had previously seen for mCHH using univariate GWAS, also in that it mainly identified loci corresponding to biologically meaningful *a priori* candidates [6,20,35].

However, while our conditional GWAS approach clearly improved the power to reject the null hypothesis of no association, interpreting the result in terms of causality is more difficult. By controlling for mCHH we have effectively simplified the trait, presumably revealing genetic factors affecting mCHG only, perhaps by affecting the maintenance of this type of DNA methylation. There is considerable background experimental evidence to support such a model. For example, the RdDM pathway would affect both mCHH and mCHG via *de novo* methylation, while mCHG (but not mCHH) would be also maintained by the CMT3 pathway, and by controlling the former, we would reveal the latter.

Our GWAS results are consistent with this interpretation. Polymorphism at *NRPE1*, a key component of the RdDM pathway, was revealed in univariate GWAS, while variation in *CMT3* (and its regulator, *MIR823a*) was only found after controlling for mCHH. At the same time, it seems clear that reality is more complex, and that both mCHH and mCHG are regulated by multiple homeostatic mechanisms that also involve factors not included in our model, like histone modifications [68]. It is therefore not surprising that most of the polymorphisms we have identified seem to affect both traits, albeit not to the same extent (exceptions include the one at *MIR823A*, which only seems to affect mCHG, and the previously identified *CMT2a’* and *CMT2b’* polymorphisms, which only affect mCHH on CMT2-targeted transposons; see S2 and S3 Figs).

Two other GWAS models produced consistent results, but had less power (S12 and S13 Figs). A fully parameterized multi-trait mixed model for mCHH and mCHG (MTMM; see [29]) confirmed that the new associations presented in this paper were “specific” to one of the two traits, while a conditional model for mCHH while controlling for mCHG (mCHH_|mCHG_) also identified most of these loci, plus the previously identified *CMT2* associations [6,35], which affect mCHH only.

In conclusion, we agree with Stephens [31] that multi-trait association methods can have much greater power than univariate methods, but require an appropriate statistical framework for interpretation. However, in a model organism like *A*. *thaliana*, further experiments should guide the construction of such a framework.

### Functional importance of non-CG transposon methylation

While genomics has revealed striking geographic variation in DNA methylation, and an equally striking genetic architecture underlying this, the functional importance of all this variation has been rather less clear. Based on decades of molecular biology studies, it is reasonable to speculate that it must play some role in regulating transposon activity [5,27,69,70]. Non-CG methylation is part of a redundant system with strong epistasis between mCHH and mCHG in keeping transposons transcriptionally silent [10,28,49]. However, direct evidence based on transposon mobility in nature has been missing. Now, the evidence is gathering that the allelic variants responsible for transposon methylation variation do indeed affect transposon mobility. Baduel et al [22] showed that the *NRPE1’* polymorphism identified as affecting mCHH on RdDM-targeted transposons by Sasaki et al [35] is associated with recent transposon mobility, and we show here that the multiple loci controlling mCHG appear to play a similar role. Our findings connect complex genome defense systems proposed in molecular biology studies with natural populations. Furthermore, the geographic distribution of the relevant multi-locus genotypes suggests that selection may be acting to maintain the appropriate level of transposon silencing—for unknown reasons. Further studies, such as crosses with new allele combinations and common garden experiments, will be required to address these questions.

## Materials and methods

### Analyzed data sets

#### DNA methylation data

The DNA methylation data sets are summarized in S5 Table. Briefly, we analyzed a bisulfite-sequencing data set published in the 1001 epigenome project [20], 85 epigenetic-related mutants [27], and our own sequence data described below. All reads were mapped on the appropriate pseudo-genome provided by the 1001 genome project (1001 Genomes Consortium., 2016) using a Methylpy pipeline v1.2 [71]. DNA methylation levels were estimated as weighted methylation levels for each transposon defined in Araport11 annotation. CMT2- and RdDM-targeted transposons were defined as having differential levels of methylation (> 0.1) between wild-type and *cmt2* or *drm1drm2* in Col-0 previously described in [20]. For each line, average DNA methylation was calculated using all transposons for which at least one read was mapped.

#### RNA-seq data

The RNA-seq data are summarized in S5 Table. Quality control and adapter trimming of all RNA-seq data were conducted using FASTP [72]. Adapter trimmed reads were aligned on the reference genome Araport11 [73] using STAR v2.7 [74] with default settings. For all annotated genes, mapped read counts were calculated using featureCounts v2.0.1 in the Subread package [75], accepting reads that were mapped on multiple genes (option -M). Using the count data, differentially expressed genes were detected by edgeR v3.1 [76] with glmQLFTest() function (FDR < 0.05). For transposon expression, reads were mapped as described above but allowing multiple hits (STAR—outFilterMultimapNmax 100 and—winAnchorMultimapNmax 100). Mapped reads were calculated by TEtoolkits [77] using the TEcount() function, and differentially expressed transposons were detected using edgeR. *CMT3* expression in natural populations were downloaded from the 1001 epigenome project data [20].

### Statistics

#### Genome-wide association studies

Univariate and conditional GWAS were carried out using LIMIX [78] version 3.0.4 with a full genome SNP matrix for 774 lines [20] from the 1001 genome project (10,709,949 SNPs) and the following linear mixed model,

Y=αL+βΧ+g+e (1)

$$var(Y)=\sigma_{g}^{2}K+\sigma_{e}^{2}I$$ (2)

where *Y* is the *n*×1 vector of a phenotype, fixed terms, *L* and *X*, are the *n*×1 vectors corresponding to a covariate and a genotype to be tested (SNP) with the parameters *α* and *β*, respectively. $g∼N(0,\sigma_{g}^{2}K)$ and $e∼N(0,\sigma_{e}^{2}I)$ are random terms, including the identity-by-state kinship matrix *K* representing the genetic relatedness [79,80] and the residuals, respectively. *Y*, *L*, and *X* were z-scored. Univariate models without cofactor take *α* = 0. SNPs that satisfied minor allele frequency (MAF) > 5% were used for association studies. Bonferroni-correction was used for multiple-testing correction after excluding all SNPs less than MAF 5%. Multi-trait linear mixed models (S12 Fig) were performed to identify common genetic effects (common) and differing genetic effects (specific) between two traits using LIMIX [78] following models described in [29].

#### Enrichment

To assess the amount of signal in the data, we used the enrichment approach introduced in [37]. The rationale behind this approach is that loci that are *a priori* candidates based on independent data should be more likely to be associated than loci that are not. Enrichment thus simultaneously demonstrates that the *a priori* loci are a meaningful subset *and* that there is a real signal of causality in the GWAS. Importantly, the method relies on the empirical p-value distribution, and is insensitive to these being well-calibrated (a major advantage when dealing with population structure).

Let *x*_*p*_ be the fraction of *a priori* loci that have p-values smaller than *p*, and let *y*_*p*_ be the same fraction among the remaining loci. Note that the loci do not have to be genes, but can also be genomic regions (e.g. coding vs non-coding), and that the method is not limited to p-values, but can be used for any ordinal statistic. Enrichment is then *x*_*p*_/*y*_*p*_. If desired, significance of the observed enrichment can be assessed using a suitable permutation scheme, like the “genome rotation” method introduced in [81].

In this study, we focused on genes, and assigned each gene a p-value using the strongest SNP (MAF > 5%) association within 15 kb (upstream and downstream). Our *a priori* set was the 80 epigenetic regulators from [20].

As described in [37], the enrichment can also be used to calculate an upper bound for the False Discovery Rate (FDR) among the a *priori* loci by assuming that all non *a priori* associations are false (if this is false, the true FDR will be lower, hence it is an upper bound). The FDR is calculated as 1−(*x*−*y*)/*x* = *y*/*x*, and we emphasize again that it *only* applies to the *a priori* set of loci.

#### Heritability

SNP-heritability was estimated using REML implemented in mixmogam (https://github.com/bvilhjal/mixmogam/blob/master/phenotypeData.py). The proportion of phenotypic variation explained by identified genetic variants was calculated using *r*^2^ [82] using R scripts published in [83].

#### Correlation of the allelic effects and molecular phenotypes

For 9329 transposons common across 774 lines, differential mCHG levels induced by alleles were estimated for each transposon as the differential average methylation level between lines carrying the reference (Col-0) and the alternative allele. Differential mCHG levels induced by mutants were calculated analogously [27]. The details are described in [35].

#### Gene effect of loss-of-function mutants

Gene functions of *MIR823A* and *JMJ26* were tested using transgenic lines described below. We measured DNA methylation levels for two or three independently grown plants, and the significance of differential mean values across RdDM- and CMT2-targeted transposons was tested by Welch’s t-test (two-tailed). DNA methylation pattern of *jmj26* was compared to *ibm1* and the control samples from published data sets [27].

#### Permutation tests

We evaluated the effects of mCHG-decreasing alleles using permutation tests with 3000 randomly chosen SNP sets with the same allele frequencies as the identified alleles along the genome. For correlations between the number of mCHG-decreasing alleles and longitude or linkage disequilibrium between the five alleles and *NRPE1’* alleles, we compared the observed to the 3000 randomly chosen samples directly. For evaluation of epistatic effects between *NRPE1’* and mCHG-decreasing alleles on transposon activity, we randomly chose 3000 SNPs with the same allele frequency as *NRPE1’* and tested the effect of zero to five mCHG-decreasing alleles.

### Plant materials

Plants were grown at 21°C with a 16-h light/8-h dark cycle and humidity 60%. Whole plant rosettes were harvested individually when they reached the 9-true-leaf stage of development. The t-DNA insertion line of *jmj26* (SALKseq_069498.1) and a gamma-ray mutant *msi1-2* [53] were purchased from Nottingham Arabidopsis Stock Center (http://arabidopsis.info/) for the functional analysis. Homozygous *jmj26* was used for bisulfite sequencing after the second generation. *msi1-2* was maintained as a segregating population, and heterozygous lines were selected by genotyping and measuring *MIS1* expression by qRT-PCR.

### Transgenic lines

#### *MIR823A* CRISPR/CAS9 mutant lines

Loss-of-function mutants *mir823a* for Col-0 and 9771 were generated using CRISPR/CAS9 methods described in [84]. Briefly, to generate a vector carrying two sgRNAs targeting *MIR823A*, the target sites were incorporated into forward and reverse PCR primers, and the fragment amplified from pCBC-DT1T2 (S6 Table). Subsequently, an amplified and purified fragment was assembled using the Golden Gate reaction into pHSE401E modified to contain a mCherry marker. The resulting vector was transformed into *Agrobacterium tumefaciens* GV3101/pSOUP, then transformed into *A*. *thaliana* by the floral dip method [85]. T1 seeds were screened by mCherry marker under the fluorescence stereomicroscope (Discovery.V8, Zeiss), and the mutations were genotyped by Sanger sequencing. T2 seeds without mCherry signal were kept to amplify the stable T3 generations, which were used for further analyses.

#### Rescue analyses of *JMJ26*

The gene function of *JMJ26* was confirmed by rescue experiments. The protein-coding genomic sequence including 3 kbp promoter sequence was PCR-amplified and cloned into the pGreen0029 binary vector [86] using In-Fusion Cloning system (Takara Bio Europe, Saint-Germain-en-Laye, France) for Col-0 and DraIV 6–22 (5984) carrying the reference and the alternative allele, respectively. These constructs were used for floral dipping transformation to Col-0 background *jmj26* (SALKseq_069498.1) to create transgenic lines [85].

#### mRNA abundance

Total RNA was extracted using ZR Plant MiniPrep Kit (Zymo Research) including a DNase I treatment and was quantified by fluorometer Qubit 4 (Invitrogen). cDNA was synthesized using the SuperScript III First-Strand Synthesis System (Invitrogen) according to the manufacturer’s protocols. qRT-PCR was performed using the LightCycler 96 system (Roche) with FastStart Essential DNA Green Master (Roche). The transcript abundance was estimated by ddCT methods with an internal control *SAND* (AT2G28390) [87]. Primer information is listed in S6 Table.

### MicroRNA abundance and secondary structure

#### Material collection and RNA extraction

Siliques were collected five days after pollination when the embryos reached an early torpedo stage according to the previous report [26]. Using the tungsten needles, seeds were dissected from siliques and collected in 1.5 ml tubes, and washed 3–4 times with fresh 10% RNA later solution (Thermo Fisher Scientific). After the last wash, the buffer was replaced with 500 μl TRIzol Reagent (Ambion) and total RNA was isolated as described in [88].

#### qRT-PCR Analysis

The quantification was conducted according to the protocol in [26]. Briefly. cDNA synthesis was performed using SuperScript III System (Invitrogen), and the corresponding stem-loop primers were added to the reverse transcription reaction. qRT-PCR analysis was performed on the LightCycler 96 Instrument (Roche) with the FastStart Essential DNA Green Master (Roche). The transcript abundance was estimated by ddCT methods with an internal control *miRNA160*.

#### RNA secondary structure

RNA secondary structure was predicted by Vienna RNAFold [48] (http://rna.tbi.univie.ac.at/cgi-bin/RNAWebSuite/RNAfold.cgi) based on Minimum of Free Energy (MEF) values [89]. We applied sanger sequences of Col-0 and 9771 carrying the alternative allele for the prediction.

### RNA sequencing

RNA was extracted in the same protocol with qRT-PCR for mRNA.Total RNA was treated with the poly(A) RNA Selection Kit (Lexogen). Libraries were prepared according to the manufacturer’s protocol in NEBNext Ultra II Directional RNA Library Prep Kit (New England BioLabs) and individually indexed with NEBNext Multiplex Oligos for Illumina (New England BioLabs). The quantity and quality of each amplified library were analyzed by using Fragment Analyzer (Agilent) and HS NGS Fragment Kit (Agilent).

### Bisulfite sequencing

Genomic DNA was extracted using GeneJET Plant Genomic DNA Purification Kit (Thermo Scientific) and sheared by E220 Focused-ultrasonicator (Covaris) to target average fragments around 350 bp. Sequencing libraries were prepared using NebNext Ultra II DNA Library Prep Kit (New England BioLabs) with Methylated Adaptor (New England BioLabs). Adapter-ligated DNA was carried through the bisulfite conversion using EZ-96 DNA Methylation-Gold MagPrep Kit (Zymo Research). Bisulfite-treated samples were amplified by EpiMark Hot Start Taq DNA Polymerase and indexed with NEBNext Multiplex Oligos for Illumina (New England BioLabs). All libraries were sequenced by Illumina NextSeq550 or Hiseq2500.

## Supporting information

S1 Table Linkage disequilibrium between five loci. (XLSX)

S2 Table *a priori* genes identified by enrichment tests (FDR = 0.2). (XLSX)

S3 Table The list of genes significantly affected in *jmj26*. (XLSX)

S4 Table Lines carrying three mCHG-decreasing alleles with mCHH-decreasing *NRPE1’* allele. (XLSX)

S5 Table Key resources. (XLSX)

S6 Table Primer information. (XLSX)

S1 Fig Distribution of p-values for two GWAS models. QQ plots for univariate models for mCHG levels **(A)** and conditional models for mCHG_|mCHH_ **(B)** in RdDM-targeted transposons (left) and CMT2-targeted transposons (right).
(PDF)

S2 Fig Distribution of phenotypes and the allelic effects. The scatter plots illustrate the allelic effects on mCHH (**A** from [1]) and mCHG_|mCHH_ in RdDM- **(B)** and CMT2-targeted transposons **(C)**. Marginal phenotypic distributions for the reference and the alternative allele are plotted in blue and yellow, for univariate and conditional distributions. Red vectors show shifts of the mean value from the reference to the alternative allele in 2-dimensional phenotype space.
(PDF)

S3 Fig Comparison of the allelic effects on mCHH and mCHG. Each arrow shows the mean value shift of mCHH and mCHG levels from reference to the alternative allele. Left and right plot correspond to RdDM- and CMT2-targeted transposons, respectively. Gray arrows correspond to the genetic effects of randomly sampled SNPs, keeping the allele frequency of each tested SNP (x 3000 for each allele). Diagonal lines correspond to linear regression lines of mCHH and mCHG.
(PDF)

S4 Fig Characterization of *MIR823A*. **(A)** Sanger sequences around *MIR823A* region for lines carrying reference allele (Col-0) and the alternative allele (763, 9771). **(B)** *MIR823A* expression in a CRISPR/CAS9 *mir823a* mutant (Col-0) by qRT-PCR. Error bar shows standard deviation (*n* = 3). **(C)** Predicted secondary structure of *MIR823A* for reference (6909) and the alternative allele (9711). Secondary structures were plotted for the *MIR823A* coding region, and mountain plots were corresponding to the expanded regions, including 150 bp upstream and 200 bp downstream. **(D)** Effects of *MIR823A* on mCHH and mCG levels in RdDM and CMT2-targeted transposons in *mir823a* Col-0 and 9771 background.
(PDF)

S5 Fig The effects of *CMT2* alleles on non-CG methylation. **(A)** Genome structure of three CMT2 alleles associated with non-CG methylation variation. The CMT2 region was illustrated by mapped short-read DNA-seq data (IGV browser) for reference line (Col-0), *CMT2a’* (10018), *CMT2b’* (6969), and *CMT2c* (10023). Vertical colored lines in the IGV plots indicate SNPs. **(B)** The allelic effects on genome-wide average mCHH and mCHG levels in CMT2-targeted transposons. Only lines carrying one allele were compared with the reference line. Horizontal gray lines show median values of the reference lines.
(PDF)

S6 Fig Genetic variation around *MSI1 and ROS3*. **(A)** Zoom-in Manhattan plots (Fig 3) and the genome structure around Chr5:23553506, 23555910 (top), and 23522001 (bottom) illustrated by mapped short-read DNA-seq data (IGV browser). Vertical colored lines in the IGV plots show SNPs. **(B)** Conditional GWAS for mCHG in RdDM- and CMT2-targeted transposons. mCHH and Chr5:23555910 were both used as co-factors. Gray vertical lines indicate the Chr5:2355910 position, and horizontal lines show the genome-wide significance (p = 0.05 by Bonferroni correction). *r*^2^ was calculated from chr5:23553506 and chr5:23555910 for mCHG_RdDM_ and mCHG_CMT2_, respectively.
(PDF)

S7 Fig Characterization of *JMJ26* allele. Predicted amino acid sequences around jmjC domain of reference (6909) and the alternative allele (DraIV 6–22)(**A**) and the conserved region (**B**). Orange bars indicate nonsynonymous mutations associated with mCHG_|mCHH_ in RdDM-targeted TEs. The sequences were predicted based on polymorphism data provided by the 1001 genome project. The domain information follows the TAIR10 annotation.
(PDF)

S8 Fig Molecular phenotypes of *jmj26*. **(A)** Characterization of loss-of-function mutants, *jmj26*. Both #1 and #2 were *jmj26* homozygous lines isolated from SALKseq_069498.1 and propagated separately. WT is a segregated line carrying active *JMJ26* in the same stock. Morphology of *jmj26* and Col-0 (left) and *JMJ26* expression in leaves of *jmj26* and the wild type (right). Expression was measured by qRT-PCR. **(B)** Volcano plots show the effects on transposon transcripts highlighted RdDM- and CMT2-targeted transposons. **(C)** The scatter plot shows the effect of mCHG methylation on transposon transcripts. The gray line shows the linear regression line.
(PDF)

S9 Fig Geographical distribution of mCHG-decreasing alleles. The plots show the origin of lines carrying mCHG-increasing or decreasing alleles.
(PDF)

S10 Fig Geographical distribution of cumulative mCHG-decreasing alleles. **(A)** Longitudinal frequencies of cumulative mCHG-decreasing alleles (corresponding to five alleles in Fig 6A). **(B)** The histogram shows how the cumulative mCHG-decreasing allele number is correlated with longitude of the origins. The blue histogram shows permuted Pearson’s correlation coefficients (R) between numbers of mCHG-decreasing alleles and longitude of the origin, maintaining the allele frequencies. The permutation tests were repeated 3000 times for 971 lines ranging from longitude -20° to 100°. The orange vertical line indicates the observed value. **(C)** Histogram similarly shows permuted Pearson’s correlation coefficients between number of mCHG-decreasing alleles and *NRPE1’* genotype. The orange line is the observation. mCHH-increasing and decreasing *NRPE1’* alleles are 0 and 1, respectively. **(D)** The geographic distribution of mCHG_|mCHH_ levels in CMT2-targeted transposons.
(PDF)

S11 Fig Function of mCHG-decreasing alleles on transposon activities. **(A)** Effects of the cumulative mCHG-decreasing alleles in whole populations (left) and combination with *NRPE1’* allele (right). (+) and (-) are lines carrying *NRPE1’* reference (mCHH-increasing) and the alternative (mCHH-decreasing) alleles, respectively. **(B)** Effects of the five major mCHG-decreasing alleles with *NRPE1’* reference (left) and the alternative (right) populations. (R) and (A) are reference and the alternative alleles, respectively.
(PDF)

S12 Fig The genetic basis of mCHG and mCHH analyzed by MTMM. **(A)** Manhattan plots for any, common, specific SNP effects on mCHG and mCHH in RdDM and CMT2-targeted transposons (see Methods). Vertical lines correspond to genome-wide significance (*p* = 0.05 by Bonferroni correction). **(B)** Enrichment of *a priori* genes and FDR for each GWAS result.
(PDF)

S13 Fig Conditional GWAS for mCHH_|mCHG_. The genetic effects on mCHH in RdDM- and CMT2-targeted transposons were analyzed by the conditional GWAS model with mCHG as cofactor. Vertical lines correspond to genome-wide significance (*p* = 0.05 by Bonferroni correction). Orange arrows indicate peaks reported in previous studies as affecting mCHH ([1]). Each GWAS result was assessed by enrichment of *a priori* genes and FDR.
(PDF)
