# Supplementary material for: Conditional GWAS of non-CG transposon methylation in Arabidopsis thaliana reveals major polymorphisms in five genes
Source: PLoS Genet. 2022 Sep 9;18(9):e1010345. doi: 10.1371/journal.pgen.1010345 (PMC9491579; doi:10.1371/journal.pgen.1010345)
Supplement: S1 Fig — QQ plots for univariate models for mCHG levels (A) and conditional models for mCHG|mCHH (B) in RdDM-targeted transposons (left) and CMT2-targeted transposons (right). (PDF) [file pgen.1010345.s007.pdf]

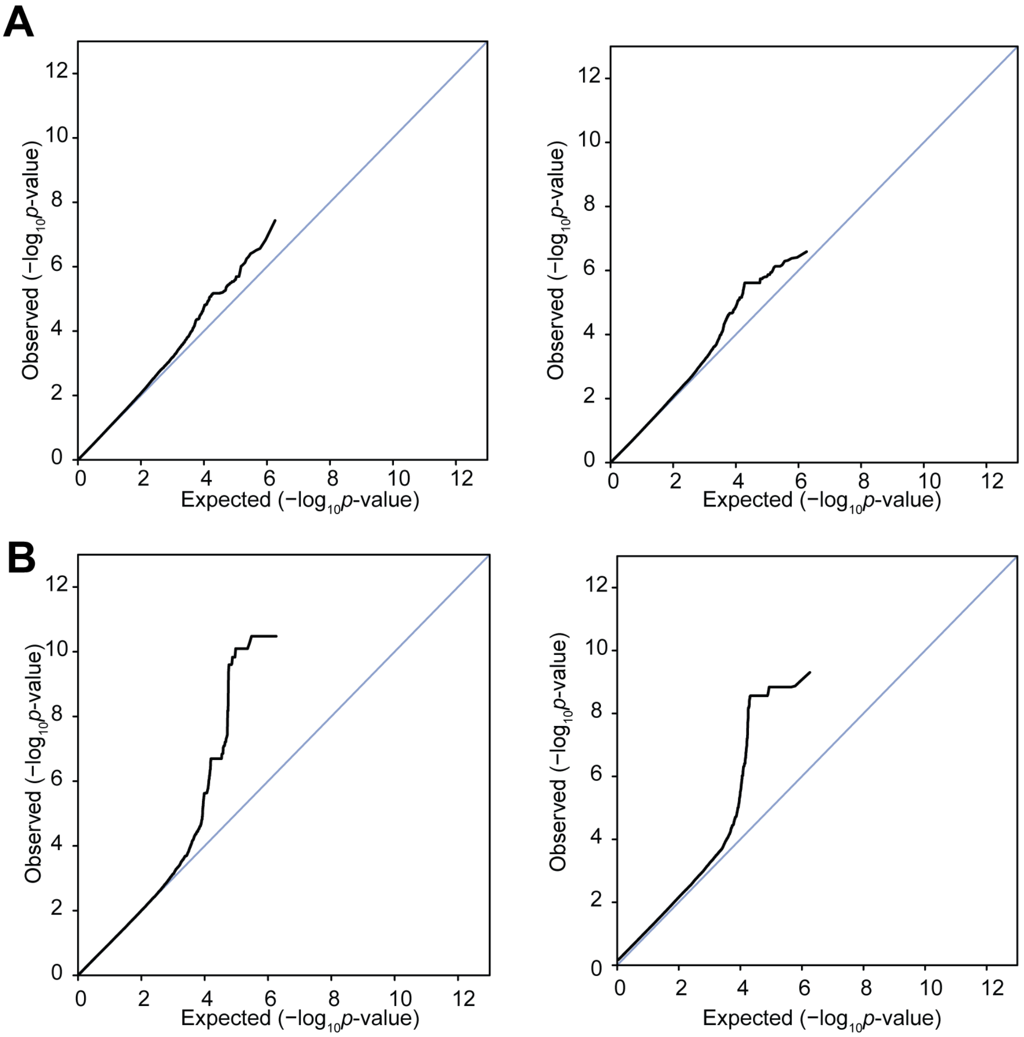

S1 Fig. Distribution of p-values for two GWAS models. QQ plots for univariate models for mCHG levels **(A)** and conditional models for $mCHG_{|mCHH}$ **(B)** in RdDM-targeted transposons (left) and CMT2-targeted transposons (right).
