# Supplementary material for: Conditional GWAS of non-CG transposon methylation in Arabidopsis thaliana reveals major polymorphisms in five genes
Source: PLoS Genet. 2022 Sep 9;18(9):e1010345. doi: 10.1371/journal.pgen.1010345 (PMC9491579; doi:10.1371/journal.pgen.1010345)
Supplement: S2 Fig — The scatter plots illustrate the allelic effects on mCHH (A from [1]) and mCHG|mCHH in RdDM- (B) and CMT2-targeted transposons (C). Marginal phenotypic distributions for the reference and the alternative allele are plotted in blue and yellow, for univariate and conditional distributions. Red vectors show shifts of the mean value from the reference to the alternative allele in 2-dimensional phenotype space. (PDF) [file pgen.1010345.s008.pdf]

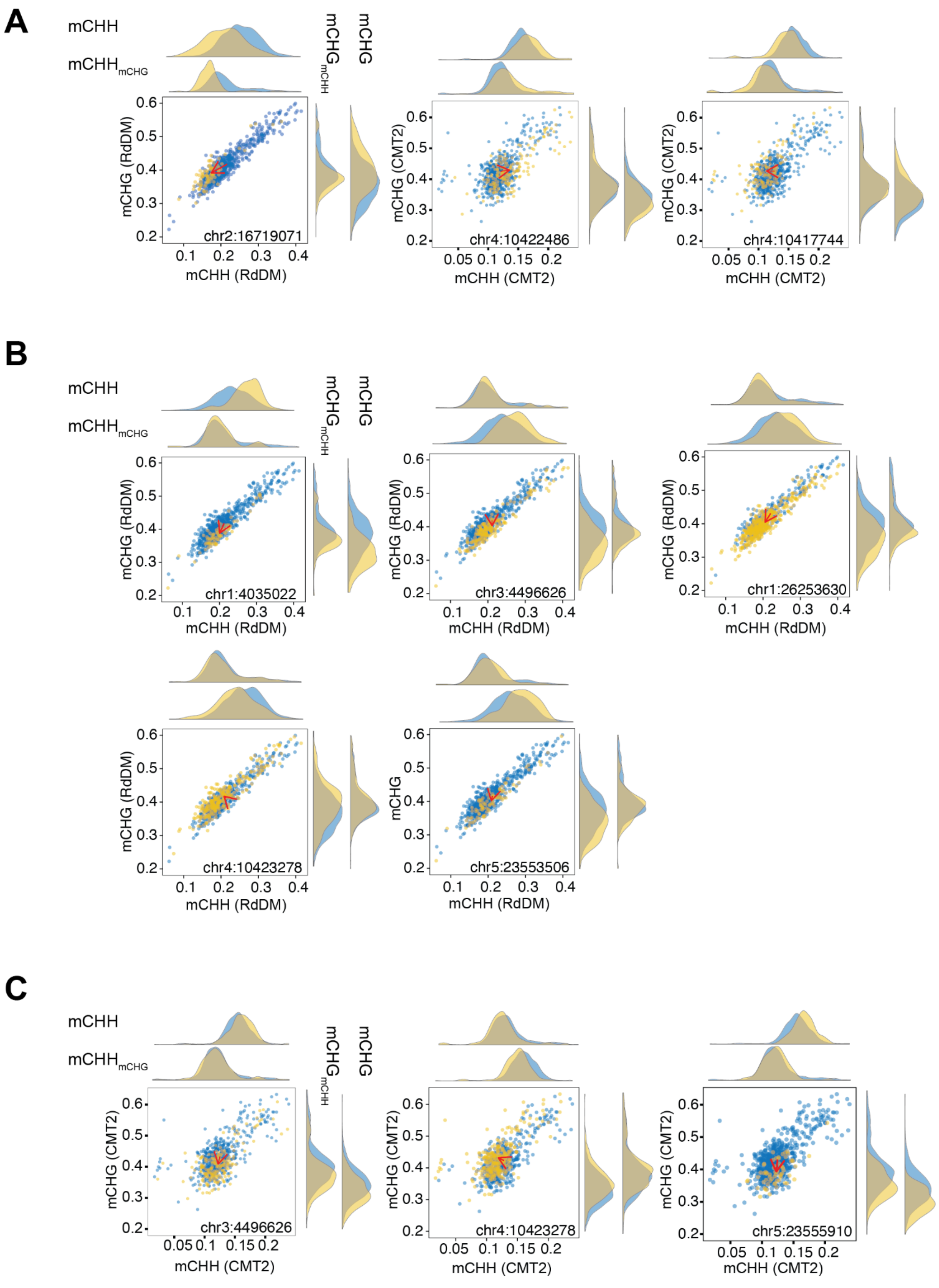

S2 Fig. Distribution of phenotypes and the allelic effects. The scatter plots illustrate the allelic effects on mCHH (**A** from [1]) and mCHG$_{|mCHH}$ in RdDM- **(B)** and CMT2-targeted transposons **(C)**. Marginal phenotypic distributions for the reference and the alternative allele are plotted in blue and yellow, for univariate and conditional distributions. Red vectors show shifts of the mean value from the reference to the alternative allele in 2-dimensional phenotype space.

Reference:

[1] Sasaki E, Kawakatsu T, Ecker JR, Nordborg M. Common alleles of CMT2 and NRPE1 are major determinants of CHH methylation variation in *Arabidopsis thaliana*. PLoS Genet. 2019;15: e1008492.
