# Supplementary material for: Conditional GWAS of non-CG transposon methylation in Arabidopsis thaliana reveals major polymorphisms in five genes
Source: PLoS Genet. 2022 Sep 9;18(9):e1010345. doi: 10.1371/journal.pgen.1010345 (PMC9491579; doi:10.1371/journal.pgen.1010345)
Supplement: S3 Fig — Each arrow shows the mean value shift of mCHH and mCHG levels from reference to the alternative allele. Left and right plot correspond to RdDM- and CMT2-targeted transposons, respectively. Gray arrows correspond to the genetic effects of randomly sampled SNPs, keeping the allele frequency of each tested SNP (x 3000 for each allele). Diagonal lines correspond to linear regression lines of mCHH and mCHG. (PDF) [file pgen.1010345.s009.pdf]

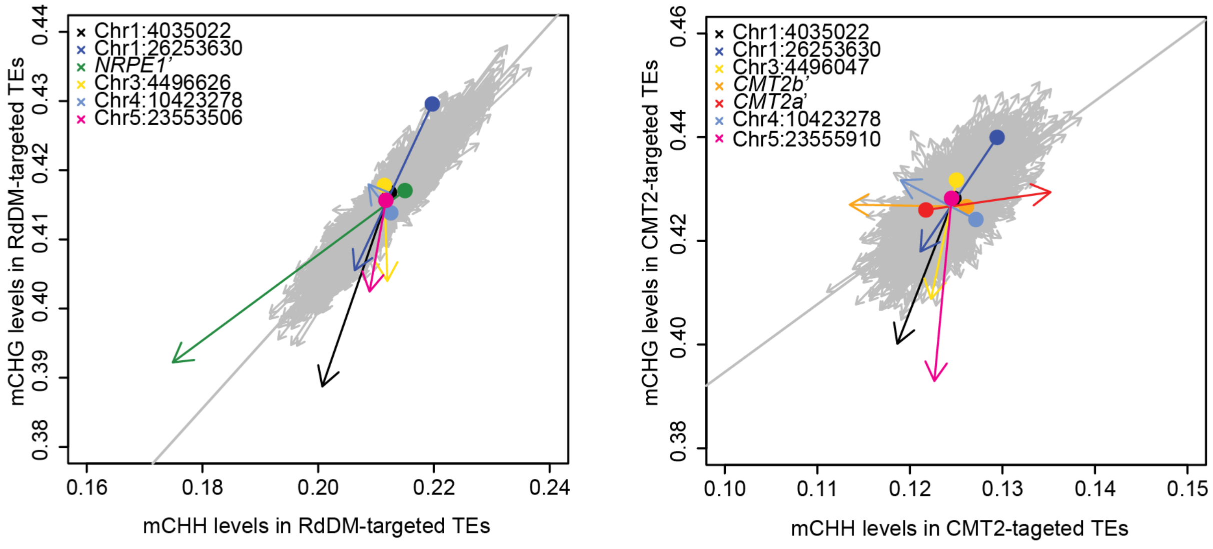

S3 Fig. Comparison of the allelic effects on mCHH and mCHG. Each arrow shows the mean value shift of mCHH and mCHG levels from reference to the alternative allele. Left and right plot correspond to RdDM- and CMT2-targeted transposons, respectively. Gray arrows correspond to the genetic effects of randomly sampled SNPs, keeping the allele frequency of each tested SNP (x 3000 for each allele). Diagonal lines correspond to linear regression lines of mCHH and mCHG.
