# Supplementary material for: Conditional GWAS of non-CG transposon methylation in Arabidopsis thaliana reveals major polymorphisms in five genes
Source: PLoS Genet. 2022 Sep 9;18(9):e1010345. doi: 10.1371/journal.pgen.1010345 (PMC9491579; doi:10.1371/journal.pgen.1010345)
Supplement: S5 Fig — (A) Genome structure of three CMT2 alleles associated with non-CG methylation variation. The CMT2 region was illustrated by mapped short-read DNA-seq data (IGV browser) for reference line (Col-0), CMT2a’ (10018), CMT2b’ (6969), and CMT2c (10023). Vertical colored lines in the IGV plots indicate SNPs. (B) The allelic effects on genome-wide average mCHH and mCHG levels in CMT2-targeted transposons. Only lines carrying one allele were compared with the reference line. Horizontal gray lines show median values of the reference lines. (PDF) [file pgen.1010345.s011.pdf]

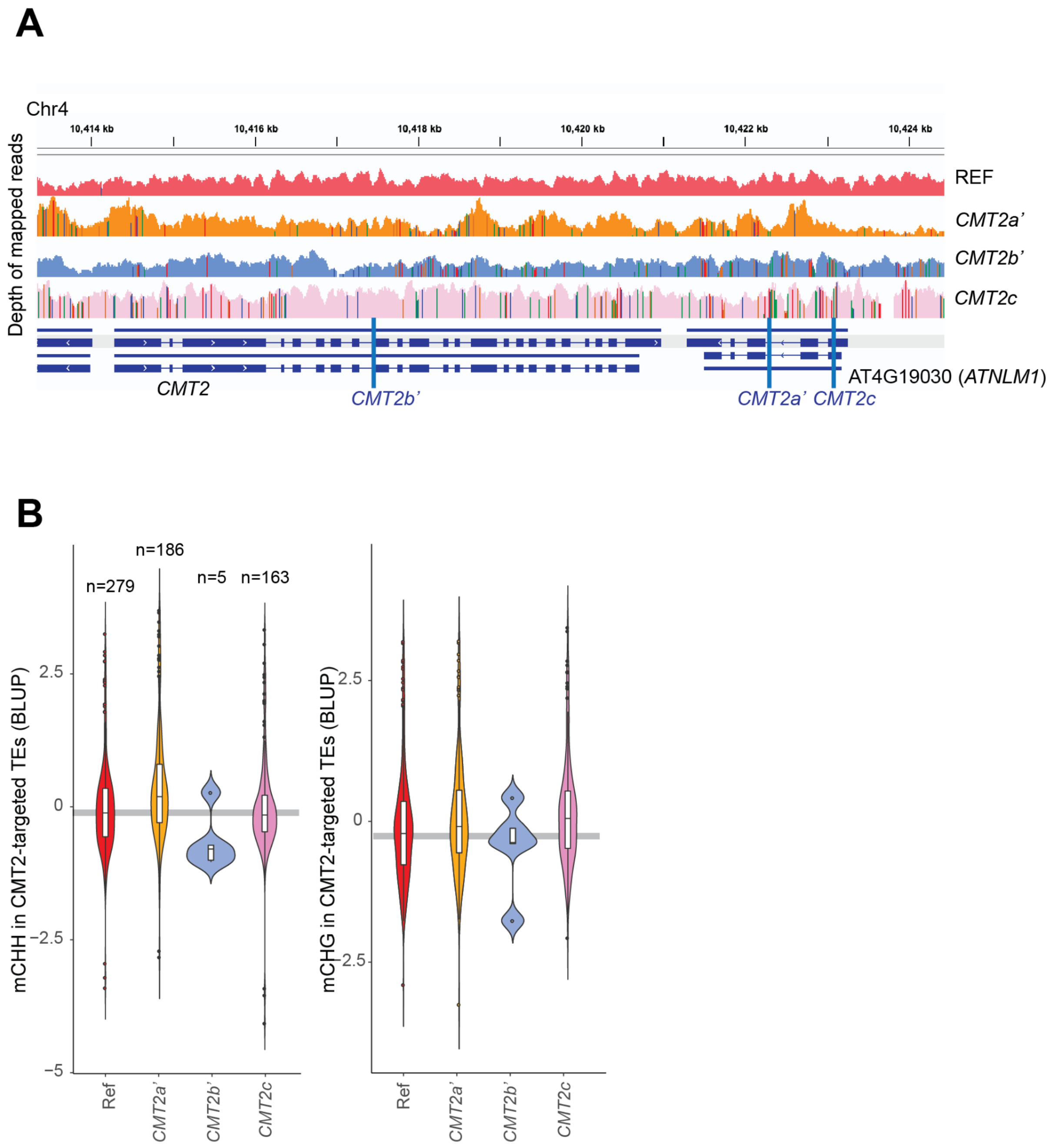

S5 Fig. The effects of *CMT2* alleles on non-CG methylation. (A) Genome structure of three CMT2 alleles associated with non-CG methylation variation. The CMT2 region was illustrated by mapped short-read DNA-seq data (IGV browser) for reference line (Col-0), *CMT2a'* (10018), *CMT2b'* (6969), and *CMT2c* (10023). Vertical colored lines in the IGV plots indicate SNPs. **(B)** The allelic effects on genome-wide average mCHH and mCHG levels in CMT2-targeted transposons. Only lines carrying one allele were compared with the reference line. Horizontal gray lines show median values of the reference lines.
