# Supplementary material for: Conditional GWAS of non-CG transposon methylation in Arabidopsis thaliana reveals major polymorphisms in five genes
Source: PLoS Genet. 2022 Sep 9;18(9):e1010345. doi: 10.1371/journal.pgen.1010345 (PMC9491579; doi:10.1371/journal.pgen.1010345)
Supplement: S6 Fig — (A) Zoom-in Manhattan plots (Fig 3) and the genome structure around Chr5:23553506, 23555910 (top), and 23522001 (bottom) illustrated by mapped short-read DNA-seq data (IGV browser). Vertical colored lines in the IGV plots show SNPs. (B) Conditional GWAS for mCHG in RdDM- and CMT2-targeted transposons. mCHH and Chr5:23555910 were both used as co-factors. Gray vertical lines indicate the Chr5:2355910 position, and horizontal lines show the genome-wide significance (p = 0.05 by Bonferroni correction). r2 was calculated from chr5:23553506 and chr5:23555910 for mCHGRdDM and mCHGCMT2, respectively. (PDF) [file pgen.1010345.s012.pdf]

A

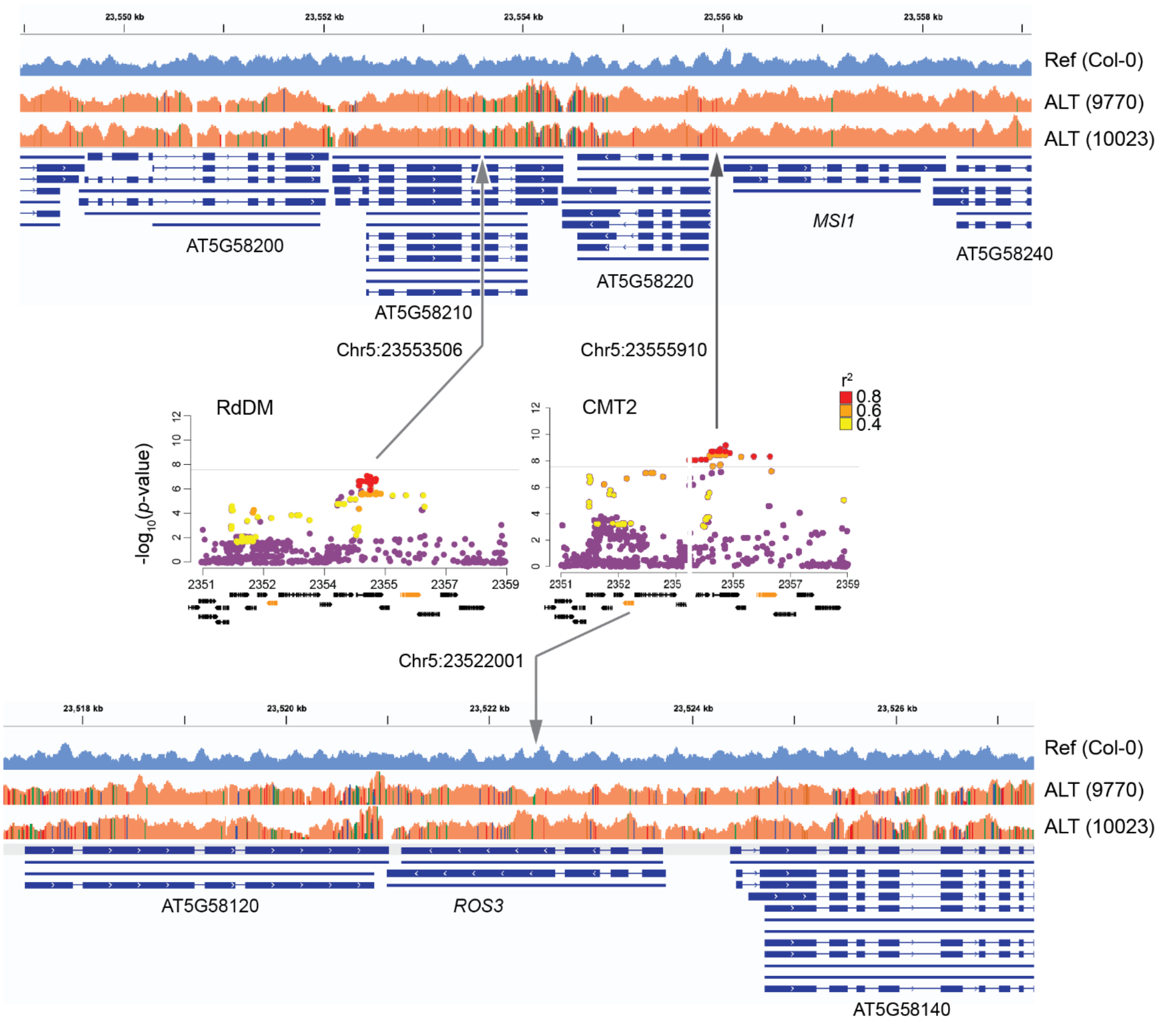

B

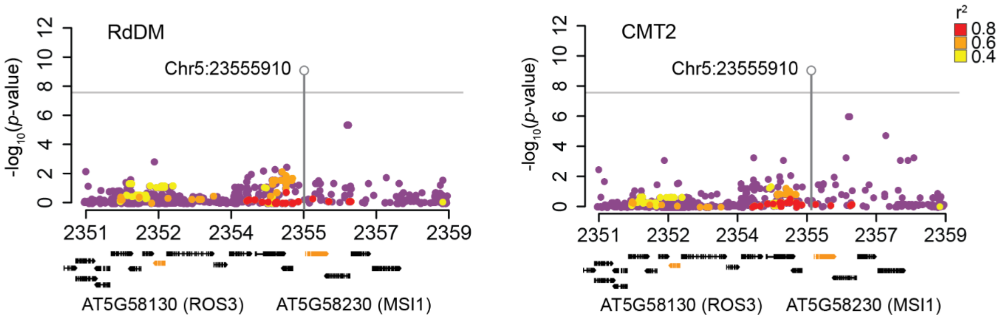

S6 Fig. Genetic variation around *MSI1 and ROS3*. (A) Zoom-in Manhattan plots (Fig 3) and the genome structure around Chr5:23553506, 23555910 (top), and 23522001 (bottom) illustrated by mapped short-read DNA-seq data (IGV browser). Vertical colored lines in the IGV plots show SNPs. **(B)** Conditional GWAS for mCHG in RdDM- and CMT2-targeted transposons. mCHH and Chr5:23555910 were both used as co-factors. Gray vertical lines indicate the Chr5:2355910 position, and horizontal lines show the genome-wide significance (p=0.05 by Bonferroni correction). r^2 was calculated from chr5:23553506 and chr5:23555910 for $mCHG_{RdDM}$ and $mCHG_{CMT2}$, respectively.
