# Supplementary material for: Conditional GWAS of non-CG transposon methylation in Arabidopsis thaliana reveals major polymorphisms in five genes
Source: PLoS Genet. 2022 Sep 9;18(9):e1010345. doi: 10.1371/journal.pgen.1010345 (PMC9491579; doi:10.1371/journal.pgen.1010345)
Supplement: S7 Fig — Predicted amino acid sequences around jmjC domain of reference (6909) and the alternative allele (DraIV 6–22)(A) and the conserved region (B). Orange bars indicate nonsynonymous mutations associated with mCHG|mCHH in RdDM-targeted TEs. The sequences were predicted based on polymorphism data provided by the 1001 genome project. The domain information follows the TAIR10 annotation. (PDF) [file pgen.1010345.s013.pdf]

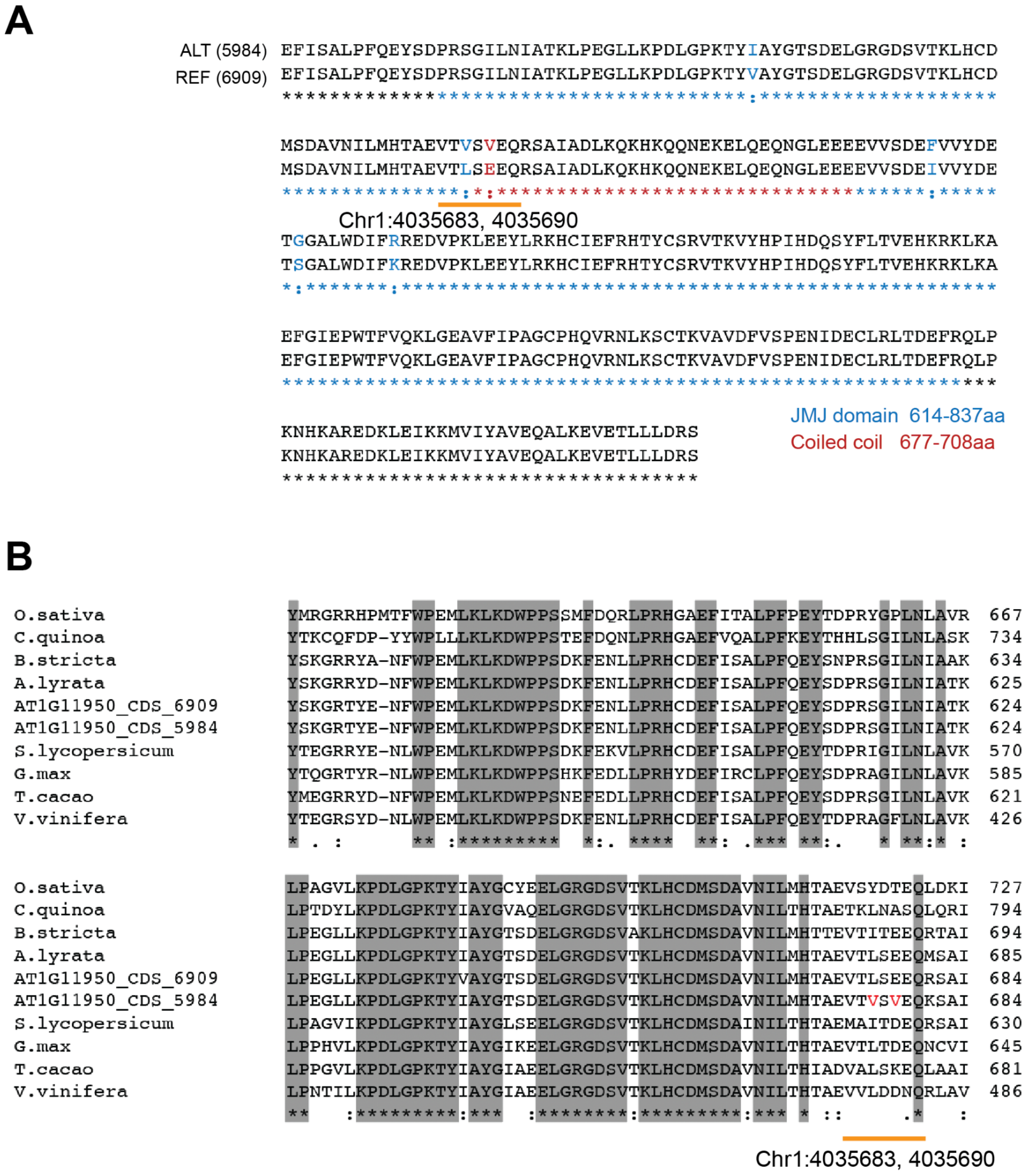

S7 Fig. Characterization of *JMJ26* allele. Predicted amino acid sequences around jmjC domain of reference (6909) and the alternative allele (DraIV 6-22) (**A**) and the conserved region (**B**). Orange bars indicate nonsynonymous mutations associated with $mCHG_{|mCHH}$ in RdDM-targeted TEs. The sequences were predicted based on polymorphism data provided by the 1001 genome project. The domain information follows the TAIR10 annotation.
