# Supplementary material for: Conditional GWAS of non-CG transposon methylation in Arabidopsis thaliana reveals major polymorphisms in five genes
Source: PLoS Genet. 2022 Sep 9;18(9):e1010345. doi: 10.1371/journal.pgen.1010345 (PMC9491579; doi:10.1371/journal.pgen.1010345)
Supplement: S8 Fig — (A) Characterization of loss-of-function mutants, jmj26. Both #1 and #2 were jmj26 homozygous lines isolated from SALKseq_069498.1 and propagated separately. WT is a segregated line carrying active JMJ26 in the same stock. Morphology of jmj26 and Col-0 (left) and JMJ26 expression in leaves of jmj26 and the wild type (right). Expression was measured by qRT-PCR. (B) Volcano plots show the effects on transposon transcripts highlighted RdDM- and CMT2-targeted transposons. (C) The scatter plot shows the effect of mCHG methylation on transposon transcripts. The gray line shows the linear regression line. (PDF) [file pgen.1010345.s014.pdf]

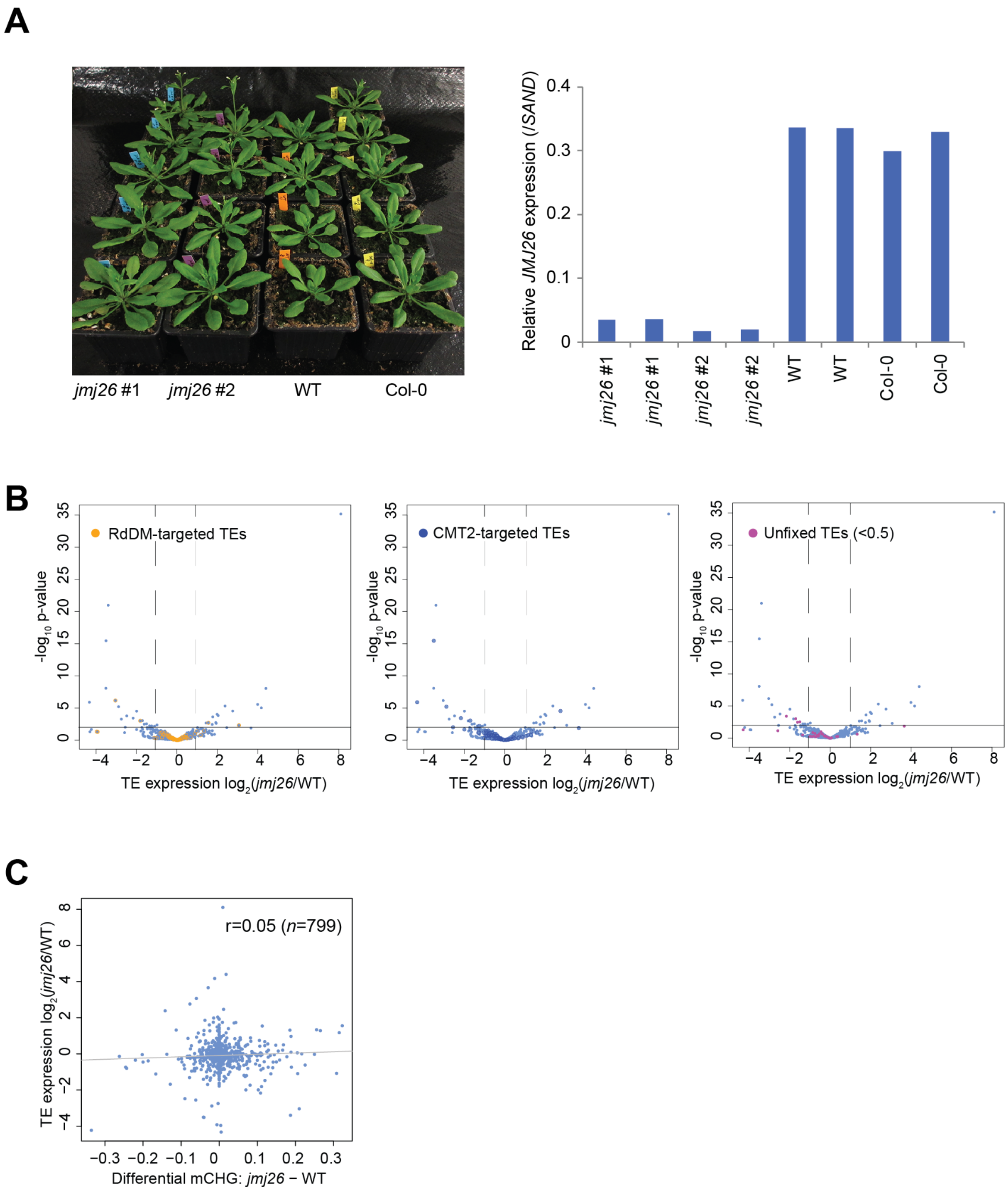

S8 Fig. Molecular phenotypes of *jmj26*. (A) Characterization of loss-of-function mutants, *jmj26*. Both #1 and #2 were *jmj26* homozygous lines isolated from SALKseq_069498.1 and propagated separately. WT is a segregated line carrying active *JMJ26* in the same stock. Morphology of *jmj26* and Col-0 (left) and *JMJ26* expression in leaves of *jmj26* and the wild type (right). Expression was measured by qRT-PCR. **(B)** Volcano plots show the effects on transposon transcripts highlighted RdDM- and CMT2-targeted transposons. **(C)** The scatter plot shows the effect of mCHG methylation on transposon transcripts. The gray line shows the linear regression line.
