# Supplementary material for: Conditional GWAS of non-CG transposon methylation in Arabidopsis thaliana reveals major polymorphisms in five genes
Source: PLoS Genet. 2022 Sep 9;18(9):e1010345. doi: 10.1371/journal.pgen.1010345 (PMC9491579; doi:10.1371/journal.pgen.1010345)
Supplement: S9 Fig — The plots show the origin of lines carrying mCHG-increasing or decreasing alleles. (PDF) [file pgen.1010345.s015.pdf]

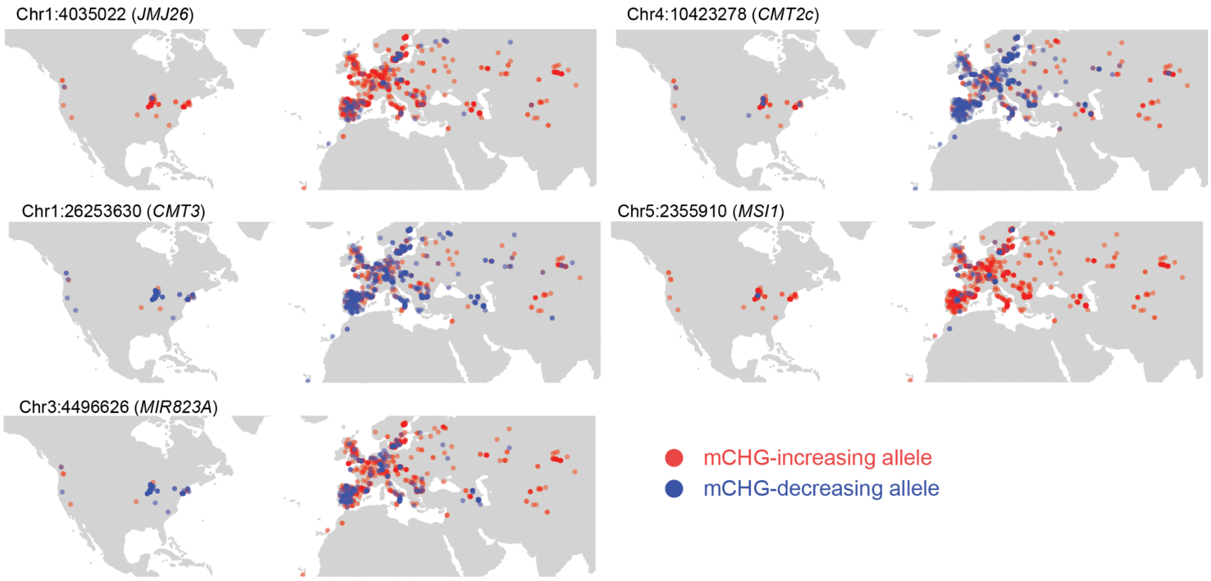

S9 Fig. Geographical distribution of mCHG-decreasing alleles. The plots show the origin of lines carrying mCHG-increasing or decreasing alleles. Mapping and statistical testing were performed in R version 3.5.3. Maps were generated using the maps package.
