# Supplementary material for: Conditional GWAS of non-CG transposon methylation in Arabidopsis thaliana reveals major polymorphisms in five genes
Source: PLoS Genet. 2022 Sep 9;18(9):e1010345. doi: 10.1371/journal.pgen.1010345 (PMC9491579; doi:10.1371/journal.pgen.1010345)
Supplement: S10 Fig — (A) Longitudinal frequencies of cumulative mCHG-decreasing alleles (corresponding to five alleles in Fig 6A). (B) The histogram shows how the cumulative mCHG-decreasing allele number is correlated with longitude of the origins. The blue histogram shows permuted Pearson’s correlation coefficients (R) between numbers of mCHG-decreasing alleles and longitude of the origin, maintaining the allele frequencies. The permutation tests were repeated 3000 times for 971 lines ranging from longitude -20° to 100°. The orange vertical line indicates the observed value. (C) Histogram similarly shows permuted Pearson’s correlation coefficients between number of mCHG-decreasing alleles and NRPE1’ genotype. The orange line is the observation. mCHH-increasing and decreasing NRPE1’ alleles are 0 and 1, respectively. (D) The geographic distribution of mCHG|mCHH levels in CMT2-targeted transposons. (PDF) [file pgen.1010345.s016.pdf]

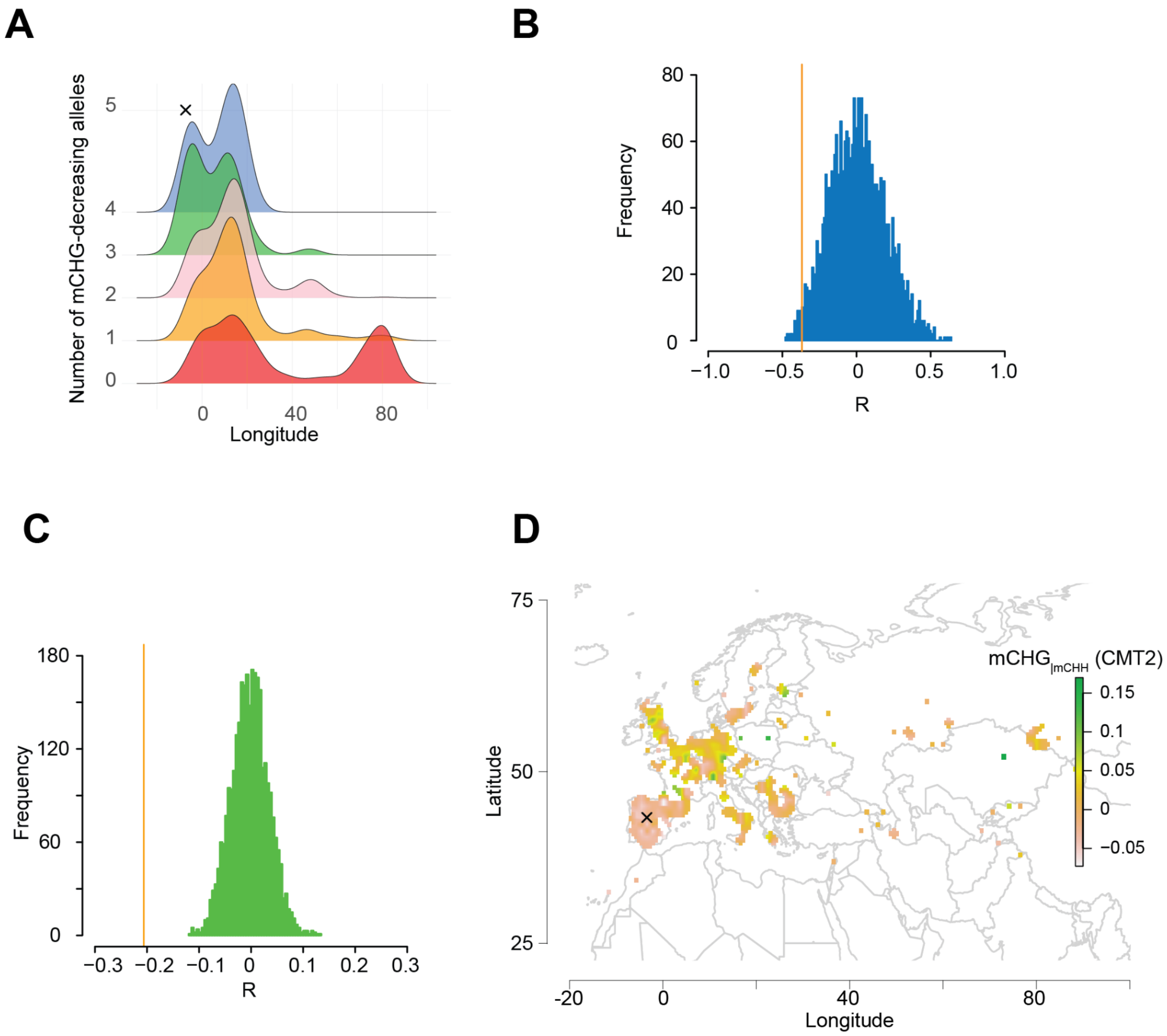

S10 Fig. Geographical distribution of cumulative mCHG-decreasing alleles. **(A)** Longitudinal frequencies of cumulative mCHG-decreasing alleles (corresponding to five alleles in Fig. 6A). **(B)** The histogram shows how the cumulative mCHG-decreasing allele number is correlated with longitude of the origins. The blue histogram shows permuted Pearson's correlation coefficients (R) between numbers of mCHG-decreasing alleles and longitude of the origin, maintaining the allele frequencies. The permutation tests were repeated 3000 times for 971 lines ranging from longitude -20º to 100º. The orange vertical line indicates the observed value. **(C)** Histogram similarly shows permuted Pearson's correlation coefficients between number of mCHG-decreasing alleles and *NRPE1'* genotype. The orange line is the observation. mCHH-increasing and decreasing *NRPE1'* alleles are 0 and 1, respectively. **(D)** The geographic distribution of $mCHG_{|mCHH}$ levels in CMT2-targeted transposons. Mapping and statistical testing were performed in R version 3.5.3. Maps were generated using the following packages: maps, ggridges, ggplot, rasrer, tidyverse.
