# Supplementary material for: Conditional GWAS of non-CG transposon methylation in Arabidopsis thaliana reveals major polymorphisms in five genes
Source: PLoS Genet. 2022 Sep 9;18(9):e1010345. doi: 10.1371/journal.pgen.1010345 (PMC9491579; doi:10.1371/journal.pgen.1010345)
Supplement: S11 Fig — (A) Effects of the cumulative mCHG-decreasing alleles in whole populations (left) and combination with NRPE1’ allele (right). (+) and (-) are lines carrying NRPE1’ reference (mCHH-increasing) and the alternative (mCHH-decreasing) alleles, respectively. (B) Effects of the five major mCHG-decreasing alleles with NRPE1’ reference (left) and the alternative (right) populations. (R) and (A) are reference and the alternative alleles, respectively. (PDF) [file pgen.1010345.s017.pdf]

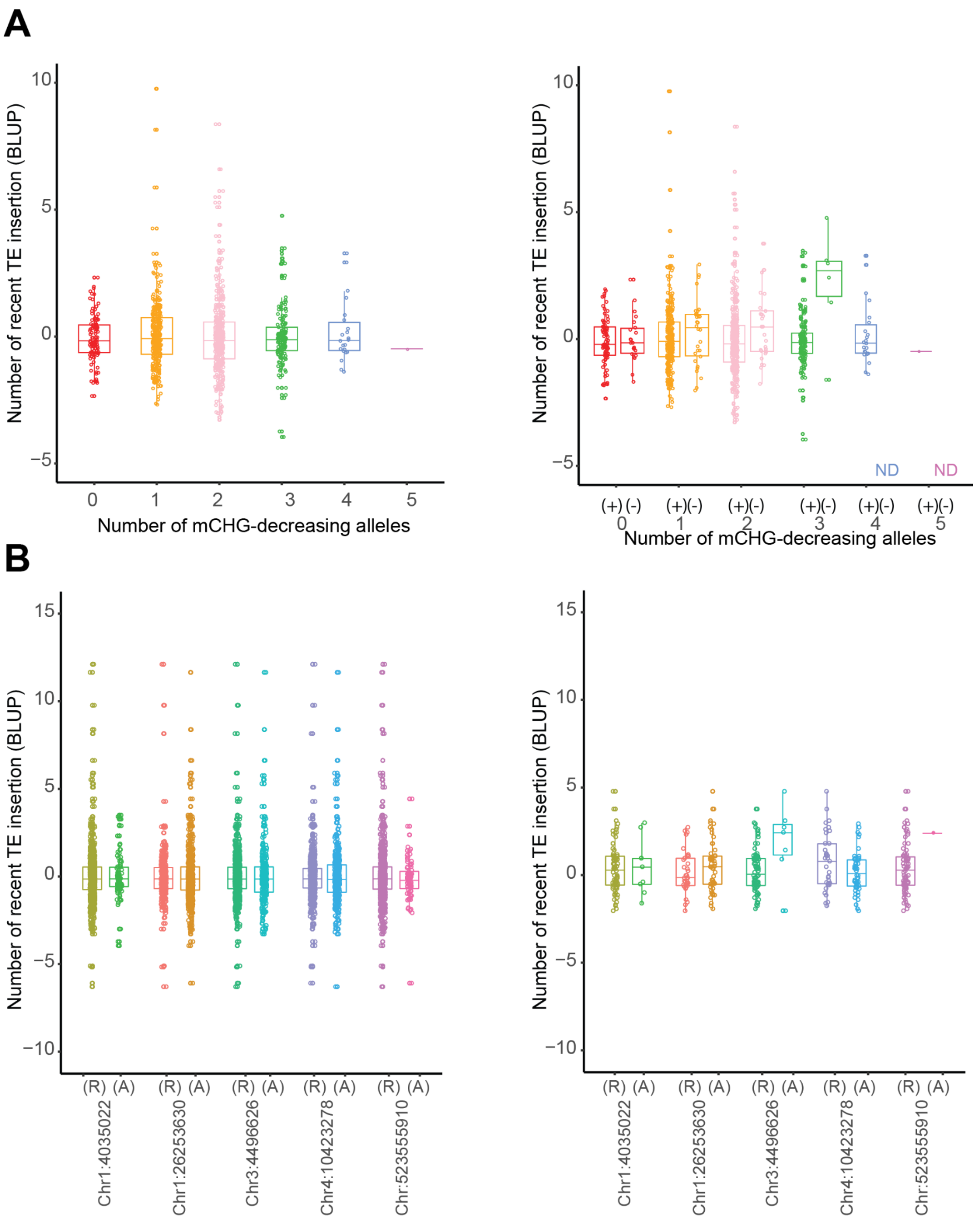

S11 Fig. Function of mCHG-decreasing alleles on transposon activities. (A) Effects of the cumulative mCHG-decreasing alleles in whole populations (left) and combination with *NRPE1'* allele (right). (+) and (-) are lines carrying *NRPE1'* reference (mCHH-increasing) and the alternative (mCHH-decreasing) alleles, respectively. **(B)** Effects of the five major mCHG-decreasing alleles with *NRPE1'* reference (left) and the alternative (right) populations. (R) and (A) are reference and the alternative alleles, respectively.
