# Supplementary material for: Conditional GWAS of non-CG transposon methylation in Arabidopsis thaliana reveals major polymorphisms in five genes
Source: PLoS Genet. 2022 Sep 9;18(9):e1010345. doi: 10.1371/journal.pgen.1010345 (PMC9491579; doi:10.1371/journal.pgen.1010345)
Supplement: S12 Fig — (A) Manhattan plots for any, common, specific SNP effects on mCHG and mCHH in RdDM and CMT2-targeted transposons (see Methods). Vertical lines correspond to genome-wide significance (p = 0.05 by Bonferroni correction). (B) Enrichment of a priori genes and FDR for each GWAS result. (PDF) [file pgen.1010345.s018.pdf]

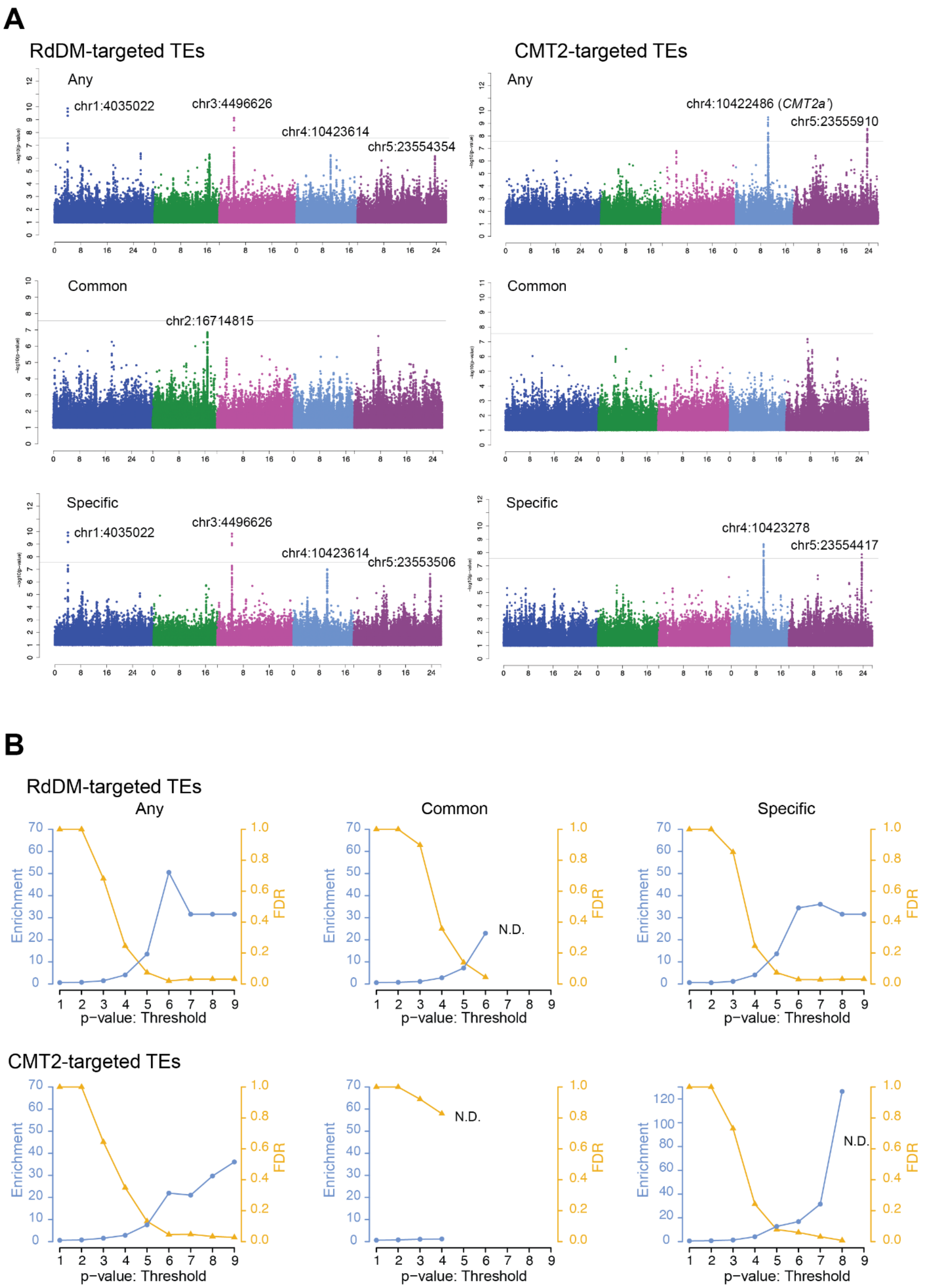

S12 Fig. The genetic basis of mCHG and mCHH analyzed by MTMM. (A) Manhattan plots for any, common, specific SNP effects on mCHG and mCHH in RdDM and CMT2-targeted transposons (see Methods). Vertical lines correspond to genome-wide significance (*p*=0.05 by Bonferroni correction). **(B)** Enrichment of *a priori* genes and FDR for each GWAS result.
