# Supplementary material for: Conditional GWAS of non-CG transposon methylation in Arabidopsis thaliana reveals major polymorphisms in five genes
Source: PLoS Genet. 2022 Sep 9;18(9):e1010345. doi: 10.1371/journal.pgen.1010345 (PMC9491579; doi:10.1371/journal.pgen.1010345)
Supplement: S13 Fig — The genetic effects on mCHH in RdDM- and CMT2-targeted transposons were analyzed by the conditional GWAS model with mCHG as cofactor. Vertical lines correspond to genome-wide significance (p = 0.05 by Bonferroni correction). Orange arrows indicate peaks reported in previous studies as affecting mCHH ([1]). Each GWAS result was assessed by enrichment of a priori genes and FDR. (PDF) [file pgen.1010345.s019.pdf]

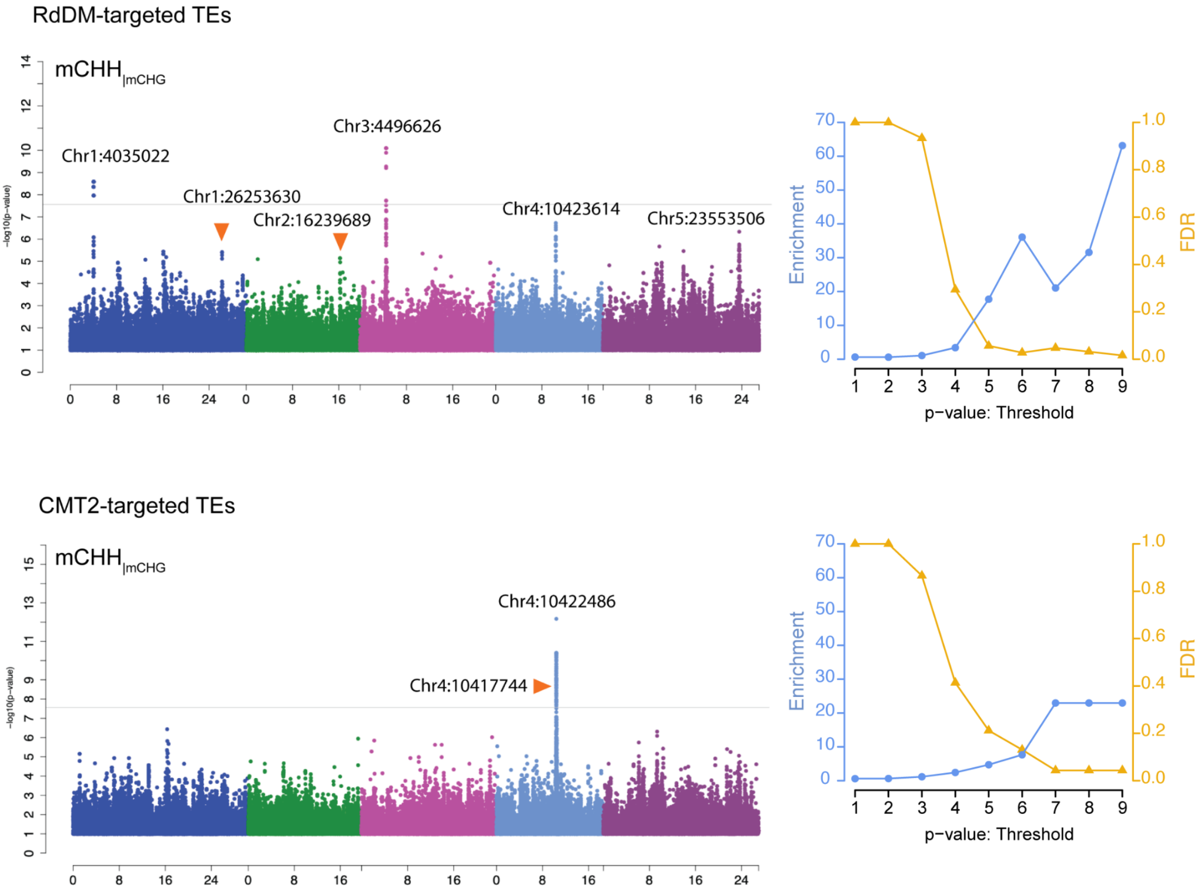

S13 Fig. Conditional GWAS for $mCHH_{|mCHG}$. The genetic effects on mCHH in RdDM- and CMT2-targeted transposons were analyzed by the conditional GWAS model with mCHG as cofactor. Vertical lines correspond to genome-wide significance (*p*=0.05 by Bonferroni correction). Orange arrows indicate peaks reported in previous studies as affecting mCHH [1]. Each GWAS result was assessed by enrichment of *a priori* genes and FDR.

Reference:

[1] Kawakatsu T, Huang S-SC, Jupe F, Sasaki E, Schmitz RJ, Urich MA, et al. Epigenomic Diversity in a Global Collection of *Arabidopsis thaliana* Accessions. Cell. 2016;166: 492–505.
