# Supplementary material for: Conditional GWAS of non-CG transposon methylation in Arabidopsis thaliana reveals major polymorphisms in five genes
Source: PLoS Genet. 2022 Sep 9;18(9):e1010345. doi: 10.1371/journal.pgen.1010345 (PMC9491579; doi:10.1371/journal.pgen.1010345)
Supplement: S4 Fig — (A) Sanger sequences around MIR823A region for lines carrying reference allele (Col-0) and the alternative allele (763, 9771). (B) MIR823A expression in a CRISPR/CAS9 mir823a mutant (Col-0) by qRT-PCR. Error bar shows standard deviation (n = 3). (C) Predicted secondary structure of MIR823A for reference (6909) and the alternative allele (9711). Secondary structures were plotted for the MIR823A coding region, and mountain plots were corresponding to the expanded regions, including 150 bp upstream and 200 bp downstream. (D) Effects of MIR823A on mCHH and mCG levels in RdDM and CMT2-targeted transposons in mir823a Col-0 and 9771 background. (PDF) [file pgen.1010345.s010.pdf]

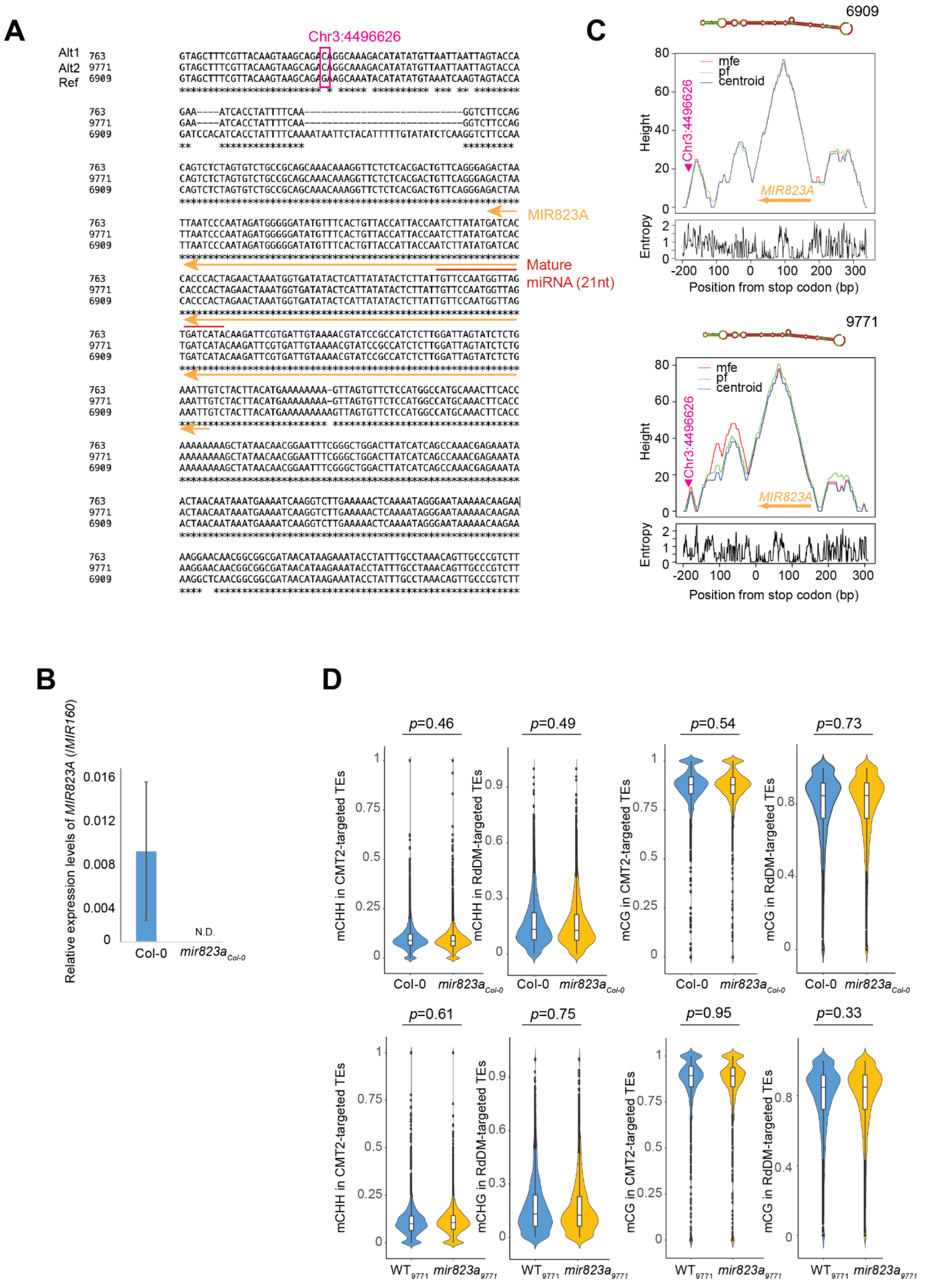

S4 Fig. Characterization of *MIR823A*. (A) Sanger sequences around *MIR823A* region for lines carrying reference allele (Col-0) and the alternative allele (763, 9771). **(B)** *MIR823A* expression in a CRISPR/CAS9 *mir823a* mutant (Col-0) by qRT-PCR. Error bar shows standard deviation (*n*=3). **(C)** Predicted secondary structure of *MIR823A* for reference (6909) and the alternative allele (9711). Secondary structures were plotted for the *MIR823A* coding region, and mountain plots were corresponding to the expanded regions, including 150 bp upstream and 200 bp downstream. **(D)** Effects of *MIR823A* on mCHH and mCG levels in RdDM and CMT2-targeted transposons in *mir823a* Col-0 and 9771 background.
