# Supplementary material for: Conditional GWAS of non-CG transposon methylation in Arabidopsis thaliana reveals major polymorphisms in five genes
Source: PLoS Genet. 2022 Sep 9;18(9):e1010345. doi: 10.1371/journal.pgen.1010345 (PMC9491579; doi:10.1371/journal.pgen.1010345)
Supplement: Attachment — Submitted filename: Cover letter.pdf [file pgen.1010345.s021.pdf]

Nathan M. Springer
Associate Editor
PLOS Genetics

GREGOR MENDEL INSTITUTE OF MOLECULAR PLANT BIOLOGY

RE: Revision of PGENETICS-D-22-00735

Vienna, Jul 7, 2022

Dear Nathan:

Many thanks for your rapid turn-around. We are glad that you liked our paper. Your request to say a bit more about likely functional changes is one we often get: we are generally reluctant to do so, because it typically amounts to pure speculation in lieu of experimental evidence. However, it is a reasonable request—like I said, we are often asked this question—and we have added to the descriptions of the associated genes. *MIR823A* was already discussed in detail, but we now note that the top association for *CMT3* is 50 bp upstream in a potential regulatory region; that the top association for *MSI1* is presumably also regulatory, as it is 102 bp upstream; but that *JMJ26* has two very strong associations that lead to predicted amino acid changes in the conserved JMJ domain, and could well be causal. We do not say anything about *CMT2*, as some alleles have been published previously and the new association we find here is 2 kb downstream, and probably a “synthetic association”. As noted in the paper, all bets are off when there is extensive allelic heterogeneity at a locus. Hopefully this is sufficient — thanks for the suggestion.

On behalf of all the authors,

Dr. Magnus Nordborg, Scientific Director
Gregor Mendel Institute of Molecular Plant Biology GmbH, Dr. Bohr-Gasse 3, 1030 Vienna, Austria
T: +43 1 79044 9000, E: magnus.nordborg@gmi.oeaw.ac.at, Web: www.gmi.oeaw.ac.at
Unicredit Bank Austria AG, IBAN: AT57 1100 0012 7042 7600, BIC: BKAUATWW
Commercial Court, Vienna: FN 203743y, VAT#: ATU 51438706

ÖAW AUSTRIAN ACADEMY OF SCIENCES
