# Supplementary material for: Conditional GWAS of non-CG transposon methylation in Arabidopsis thaliana reveals major polymorphisms in five genes
Source: PLoS Genet. 2022 Sep 9;18(9):e1010345. doi: 10.1371/journal.pgen.1010345 (PMC9491579; doi:10.1371/journal.pgen.1010345)
Supplement: Attachment — Submitted filename: 124661_1_rebuttal_2374828_rcql0d.docx [file pgen.1010345.s020.docx]

**Manuscript number:** RC-2022-01296

**Corresponding author(s):** Magnus Nordborg, Eriko Sasaki

## 1. Statements [optional]

*This section is optional. Insert here any general statements you wish to make about the goal of the study or about the reviews.*

We wish to thank both reviewers for their efforts. Reviewing takes time, and although we did not agree with all comments, there is no doubt that our article has improved as a result of responding to them. Neither reviewer had major criticisms. Reviewer 1 provided a number of specific suggestions for improvement, which we have incorporated when feasible. Reviewer 2 did the same, but also disagreed with our emphases and discussion points. While we generally consider these things beyond the remit of the reviewer, the comments revealed that we had failed to communicate several points clearly, and also that we needed to explain some of our statistical methods better.

## 2. Point-by-point description of the revisions

*This section is mandatory.* *Please insert a point-by-point reply describing the revisions that were already carried out and included in the transferred manuscript.*

**Reviewer #1 (Evidence, reproducibility and clarity (Required)):**

The manuscript by Sasaki et al titled "Conditional GWAS of non-CG transposon methylation in Arabidopsis thaliana reveals major polymorphisms in five genes" employed conditional GWAS to identify trans-regulators of mCHG levels in Arabidopsis natural accessions, after controlling for mCHH. Using loss of function mutants for couple of these genes, the authors also tested their effects on mCHG levels.

Overall, this manuscript makes a nice contribution. I suggest the following improvements to enhance the quality of this manuscript.

Comments:

1. MSI1 has been shown to be copurified with TCX5, a component of DREAM Complex. The DREAM complex transcriptional regulates CMT3, MET1, DDM1 in a cell cycle dependent manner (ref: Yong-Qiang Ning, 2020 nature plants). Tcx5/6 double mutants have ectopic gain of TE and genic mCHG. It would be nice to refer this paper and add to the MSI1 part accordingly.

Absolutely: thanks for suggesting this!

2. Multifaceted regulation of mCHG levels seems to be evident from this and previous studies. Why would such complex pathways evolv to regulate mCHG? Bewick et al 2016 and Wendte et al 2019 showed lack of CMT3 or ectopic expression of CMT3 can influence CG gene body methylation (gbM). One possibility is that these five factors regulate CHG to maintain it at a level that is just enough to target TE. Irrespective of the functional relevance of gbM, differences in the levels of these five factors might result in erroneous gbM. It would be interesting to look for the rates of gbM and number of gbM genes in the natural accession carrying 1 to 4 number of mCHG-decreasing alleles. Also, in the one line from Iberian peninsula carrying polymorphisms in all five genes.

Yes, the connection between CHG and gbM is very interesting and deserves more attention. We looked for the effect of cumulative mCHG-decreasing alleles on gbM, but there was no association with gbM — but this is really not expected given the stable epigenetic inheritance of gbM. The Iberian peninsula line carrying all decreasing alleles did slightly lower gbM levels, but it is impossible to exclude the effects of population structure. Since we have nothing to add beyond speculation, we prefer not to go into this topic.

3. The authors mentioned a significant peak for mCHG|mCHH on RdDM-targeted transposons was located 196 bp downstream of MIR823a and not on mature miRNA. Therefore, this cannot directly impair miR823 base pairing with CMT3 mRNA transcripts and its cleavage. Moreover, natural accessions carrying alternative MIRNA823 allele show reduced CMT3 and mCHG levels, meaning more miR823 levels? Does this 196 downstream region contain any regulatory feature that effects miR823 transcription? Or this region still falls in the primary miRNA hairpin region? A single nucleotide change in pri-miRNA can have a significant impact on its secondary structure that can impede DICER processivity and effectively levels of mature miR823 molecules? It will be beyond the scope of this paper to pin down the exact mechanism. But a simple stem loop RT-PCR for miR823 levels in reference and alternative accessions would be informative (on accessions that grow at the same speed). Perhaps, the authors can at least model SNP induced pri-miRNA secondary structure variations using Vienna RNAFold (<http://rna.tbi.univie.ac.at/cgi-bin/RNAWebSuite/RNAfold.cgi>) and present MEF values (maximum free energy) for representative accessions.

Stem-loop qRT-PCR for *MIR823a* expression would indeed be helpful to confirm allelic effects. However, comparing lines with wildly different genetic backgrounds is fraught with difficulty due to *trans*-effects. Furthermore, *MIR823a* is expressed specifically during embryogenesis, and the expression quickly decreases after the early heart stage (Papareddy et al., 2021). Thus, we would need to extract microRNA from embryos at exactly the same developmental stage, from lines that may develop at different speeds.. Most likely, time-series data would be required, and generating such data is a massive undertaking. As noted in the paper, we did measure *MIR823a* expression by stem-loop qRT-PCR for several lines carrying reference and alternative alleles but the results were inconclusive. A proper study of this is beyond the scope of this paper.

Testing predicted effects on RNA secondary structure, on the other hand, is eminently feasible. As suggested, we used Vienna RNAFold for the region, including the GWAS peak. Since the SNP is linked to a 35 bp deletion (shown in S4A), it is closer to the *MIR823A* coding region than 196 bp. However, the results indicate that the SNP (Chr3:4496626) is not within the stem-loop. It remains possible that this SNP tags multiple SNPs in the annotated stem regions. This is now mentioned.

4. Figure 1A can be made more reader friendly. Perhaps this can be broken down into correlation plots for individual conditions or tissue types. In addition, it might be good to add individual r-square values for each of them instead of compound r-square.

We respectfully disagree, since the main point of the figure is the overall correlation and heterogeneity, rather than the correlation within sub-sets. Instead of splitting the plot, we changed color contrasts to make it easier to read.

5. Page 3, Paragraph 1 from line 3 to end of paragraph. The authors wrote "Much of this variation is due to differences in the environment (including tissue, which can be viewed as a cellular environment)". A possible explanation is these two tissues have different mitotic indices (fraction of cells diving and non-diving; flowers have more dividing cell, leaves have more non dividing and endoreduplicated cells) that explains non-CG variation. I would suggest authors to change the text to this and refer to Filipe Borges et al 2021 Current biology paper.

This is certainly a possibility, although higher mCHG levels in flower buds presumably also reflect higher *CMT3* expression during embryogenesis (Feng et al. 2020; Gutzat et al. 2020; Papareddy et al. 2021). We now mention both explanations and cite Borges et al. (2021).

**Reviewer #2 (Evidence, reproducibility and clarity (Required)):**

Summary:

Sasaki et al. carried out a conditional GWAS analysis of TE-CHG methylation in Arabidopsis thaliana natural accessions. They revealed multiple associations with SNPs in known DNA methylation genes. A new finding is the association found proximal to JMJ26, which had no previously described role in the maintenance/establishment of RdDM-targeted transposons. The authors validate the JMJ26 association using a loss-of-function mutant of JMJ26, which essentially recapitulates the GWAS effect, suggesting that JMJ26 is likely causal. An important point of the study is that the associations detected with conditional GWAS have not been seen in previous univariate (i.e. unconditional) GWAS, probably due to to a lack of power. At the sub-genome-wide threshold the authors discovered further, albeit weaker, associations that were also highly enriched for known DNA methylation genes.

Overall impression:

The manuscript is clearly written, and the functional validation of the JMJ26 GWAS signal is commendable and certainly goes beyond the typical GWA study. Beyond this validated association however, the GWAS results are mainly confirmatory. They essentially highlight that methylation genes previously identified by way of mutant screens are variable in natural populations, and (probably) causative of non-CG methylation variation in TEs. What I personally found very distracting throughout the manuscript was the strong emphasis on the methodological aspect; that is, the conditional GWAS, which is really not new. Furthermore, the conceptual/philosophical discussion about what is a complex trait or what can be called polygenic was slightly pedantic and distracted from the biological message.

There are three points here. First, we disagree that the GWAS results are confirmatory. Sure, only one of our associations is connected with a novel gene, but the fact that the four other genes apparently harbor major polymorphism is a new finding that contributes to our understanding of the function of this trait (and, possibly, these genes). Second, while it is possible that we emphasize statistical methodology too much, we do this for clarity, not to claim that what we are doing is novel. Third, we are similarly not interested in defining what is polygenic and what isn’t, but rather put the results in the context of other studies. We have changed the writing in various places to make it clearer (and hopefully less distracting/pedantic).

A conceptual comment:

- The conditional GWAS presented here is conceptually very similar to conditional QTL mapping approaches where candidate loci are included, a priori, as covariates in the model, and a scan is performed to search for additional modifiers. It is known that this approach increases power because the scan is performed on the residual trait variation (having accounting for effect of candidate loci). This is also the idea behind MQM mapping, although in the latter the inclusion is not restricted to candidate loci. Instead of including candidate SNPs as covariate the authors include TE-CHH methylation levels as a covariate as it is highly correlated with TE-CHG methylation. By doing this, the authors essentially "control" for any SNP affecting the covariance between CHG and CHH, even if these SNPs (and their genetic architecture) remain unknown. Hence, the conditional scan is mainly on the residual variation in TE-CHG methylation that is unique to this context (i.e. independent of CHH). That additional TE-CHG associated loci pop up in this scan is perhaps not so surprising.

We agree, and have even written papers on this very subject. We were surprised by this comment as we felt we had included lengthy sections (see also comment above) about methodology, emphasizing that multi-trait analysis is a good idea in principle. One of our purposes here is to provide a beautiful example demonstrating this. We have tried to make these points clearer.

The finding that this conditional GWAS yields again a handful of loci of that explain a considerable part of the trait (now residual trait) variation leads the authors to suggest that the genetic architecture underlying non-CG methylation of TEs is not "polygenic". I think this is semantics. All the authors have done is relegate any causal SNPs underlying the covariance between TE-CHG and TE-CHH to the right hand side of the equation of their GWAS model, and subsumed it under the predictor "TE-CHH methylation levels". That is, the genetic architecture underlying this covariance is still unknown, difficult to identify and probably highly polygenic.

Again we agree, and fail to see why the reviewer thinks we do not. Nowhere do we claim that the overall covariance has a simple basis, and we explicitly state that it is the conditional mCHG variation that has an oligogenic basis. We did write that “univariate GWAS of mCHG variation failed to detect any significant associations, leading us to conclude, erroneously, that the trait was simply too polygenic”, which was imprecise, and arguably erroneous. The word “erroneously” has been removed in the revision.

The authors essentially decompose a complex traits into parts and map genetic architectures for each part. Although each part seems less complex and more oligogenic than polygenic, when putting all the parts back together, I would argue we are getting close to a complex trait with a polygenic architecture. The study by Hüther et al, which the authors also cite, is another example of how a complex trait can be decomposed into parts. In reference to one of the authors' GWAS associations, they say "...this association was also recently found by Hüther et al. (2022) using GWAS for unconditional mCHG levels of individual transposons. The MIR823A polymorphism appears to almost exclusively affect mCHG (Figs. S2, S3), primarily targeting the same transposons as a CMT3 knock-out...". In the case of Hüther et al., the complex TE-CHG methylation trait is simplified by selecting specific TEs, a priori, that are differential methylated in CMT3 knock-out lines. One could go on like this, and continue to peel away this complex trait. But, again, this does not mean that the overall TE-CHG methylation trait is not complex nor polygenic. It spirals down into a discussion of what is actually meant by "complex" or "polygenic", which is an interesting discussion, but - in the case - of this manuscript takes away from the biological message. My point is perhaps best reflected in the following statement from the discussion section: "Despite high heritability, univariate GWAS of mCHG variation failed to detect any significant associations, leading us to conclude, erroneously, that the trait was simply too polygenic (Kawakatsu et al., 2016)." But a few lines below the authors seem to realize what they have actually done "We believe that, by controlling for mCHH, we have effectively simplified the trait, revealing genetic factors affecting mCHG only, perhaps by affecting the maintenance of this type of DNA methylation."

The phrase “seem to realize” is unwarranted and unnecessary sarcasm. Given that we cite the two century-old papers that first demonstrated that it was possible to decompose complex traits into Mendelian ones, it should be obvious that we understand what we have done. That our writing could have been better is another matter. As noted above, the word “erroneously” has been dropped, and we have also changed the second sentence to make it obvious that this is obvious. We suspect that whether one finds this part of the Discussion “distracting” or not depends on training and background — our objective was to explain our results to readers who (unlike us and the reviewer) are not well-versed in quantitative genetics.

Specific comments

1. A large part of the manuscript focuses on SNPs that enriched for a priori genes that fall below the genome-wide significance threshold. While I see the reasons for doing this in this particular manuscript, I do not see how this is useful in general (again this approach is partly "sold" on methodological grounds). The approach can obviously not be extended to study traits where a priori gene sets are unavailable or incomplete. Moreover, the "FDR" approach based on the a priori gene set labels GWAS hits that are not within the a priori set "false discoveries", which may or may not be true. Moreover, there is no "natural" stopping point for going below GWAS thresholds. An alternative, to this would be to perform a targeted GWAS for a priori genes (+ a LD window around them). Since this alleviates the multiple testing burden, I would be curious to see what this yields both in terms of conditional and unconditional analysis. Candidates that show a signal could be included as covariates and a conditional scan for unknown genes could then be performed.

9. The FDR analysis using a set of a priori genes should be explained in detail in this ms. It is cumbersome to go to another manuscript to see what was done exactly, especially since this information is also difficult to dig up in the Atwell 2010 study. Although I understand the idea behind this approach, I would be concerned that this type of "FDR" analysis assumes that that all methylation genes are known. A novel candidate that was perhaps never identified in mutants screens before would be classified as a false discovery. Similarly, known candidates that carry no functional polymorphisms in nature, perhaps because they are highly constraint, will never become a discovery.

Comments 1 and 9 largely overlap, and so we moved 9 here for clarity and respond to both at the same time. We agree that the enrichment analysis should be explained in this article as well, so as to save the reader from finding the supplement to an old paper. A new section has been added to Methods. In this section, we also try to preempt some of the misunderstandings in the reviewer's comments.

First, our approach is indeed generally applicable. Whether it is useful depends on what you want to do, and yes, the utility will depend on the quality of the independent data, but note that the *a priori* gene set does not have to be genes: you could use this approach to compare coding vs non-coding regions of the genome, for example.

Second, we are not trying to “sell” our approach (or anything else for that matter).

Third, the approach does not label GWAS hits that are not within the *a priori* set as false discoveries: it says nothing about these hits.

Fourth, we are not sure what is meant by a ‘“natural” stopping point for going below GWAS thresholds’, but our approach does provide a simple way to explore how FDR (in the *a priori* set!) depends on the threshold used.

Fifth, the proposed alternative of “targeted GWAS” (non-genomewide association, as it were) is not equivalent, because our approach was not designed to increase power by alleviating the multiple testing burden, but rather to rigorously demonstrate that there is a signal in the data when faced with uncalibrated p-values. That it can also be used to explore sub-significant associations is a nice side-effect that we exploit here.

Sixth, we do not assume that all methylation genes are known, nor is our goal to find them all.

2. With regards to the CMT2 signals (particularly section "Further evidence for allelic heterogeneity at CMT2") it would have been useful/clearer to break down CHH into CWA and non-CWA.

While this is a sensible suggestion, the focus of this paper is on mCHG, and refining the mCHH measurement would essentially amount to re-doing all analyses.

3. I understand that the authors set out to do this conditional analysis because previously no hits could be found for CHG TE methylation. However, have the authors considered going the other way around and performing a CHH|CHG analysis to find additional QTL affecting CHH methylation, partly indepedently of CHG?

Yes, this was in the paper, but we only mention it in the Discussion (and Fig S13) as the results were only of methodological interest (as expected, they were very similar).

4. The authors write: "While both mCHG and mCG showed high heritability, GWAS yielded little in terms of significant associations. This might be because these "traits" are highly polygenic, or because they are at least partly transgenerationally inherited, and hence do not behave like standard phenotypes." Please clarify what they mean by "not behave like standard phenotypes".

Done.

5. The authors write: "Our starting point is the observation that mCHG and mCHH levels on transposons are strongly correlated in the 1001 Epigenomes data set (Kawakatsu et al., 2016), especially for RdDM- targeted transposons (Fig. 1A; see Methods). Much of this variation ....". What is mean by "this variation"?

The sentence has been changed to make this clearer.

6. A few lines below, they write "...huge". Please rephrase.

Done.

7. The authors write: "sample data set ("Leaf SALK ambient temperature"; n=846). Interestingly, the covariance between mCHH and mCHG showed the same pattern in data generated by knocking out known or potential DNA methylation regulators in the same genetic background (Fig. 1B) (Stroud et al., 2013). This demonstrates strong co-regulation of these types of methylation, in particular for RdDM-targeted transposons." It is noticeable that many double mutants are off the diagonal. To me this indicates that they affect one context more than the other (i.e. they break covariance). Second, it suggests that they are probably interacting non-additively. It would be great if the authors could comment on this observation; perhaps also later in the ms, where they make a case for additivity.

We are not convinced that the double- or triple-mutant show non-additivity. Adding up effects in Figure 1 works pretty well. As for our GWAS results, it is clear that small effects (like the ones in our GWAS) will always tend to look additive for simple mathematical reasons. This does not mean that no interactions exist, and we emphasize this in the paper. We also have an example of non-linearity when it comes to TE activity. This is now also emphasized.

8. The authors write: " it is difficult to say what fraction of these factors is genetic and what is environmental, but, regardless of this, we hypothesized that the substantial covariance could reduce power of GWAS for either mCHH or mCHG (when using a standard univariate model), and that an analysis accounting for this covariance might perform better...". The arguments given thus far are not sufficient to understand why a "substantial covariance" between traits would reduce the power to map individual traits. I think more needs to be done here to motivate this.

The sentence following the one quoted is “In essence, we sought to simplify a complex trait by breaking it into constituent parts”, which is very much part of the motivation. As the reviewer noted above, it is not surprising that a conditional analysis turns out to be more powerful. The comment may have arisen from the statement “This insight is the basis for this paper”, which is misleading — there is no insight here, just a very obvious hypothesis, which turned to be correct. We have changed the writing to make this clearer.

10. The authors write" "However, MSI1 is required to control DNA methylation via repression of MET1, and a loss of FAS2 in CAF-1 induces mCHG hypermethylation (Fig 1B) (Stroud et al., 2013; Jullien et al., 2008)...", where is the "FAS2 in CAT-1" result visible in Fig. 1B?

*fas2* induces mCHG hypermethylation in CMT2-targeted TEs, presumably via a complex that also involves *MSI1*. It is marked in Fig. 1B. We have rephrased the sentence to make this clearer.

11. The results presented in "A jmjC gene is a novel modifier of mCHG in RdDM-targeted transposons" could have been showcased better. Only after reading the methods part did I realize that the authors generated CRISPR mutants. It reads as if the authors just picked up some available loss of function mutants and profiled them. But, clearly, much more work was involved here and the authors could have brought that out more. Perhaps more generally, I think all the new functional analysis the authors perform is largely "under-sold" in this manuscript at the expense of unnecessary methodological/concpetual discussion (see point above).

We actually generated CRISPR/CAS9 mutants only for *MIR823A* (Table S5). For *JMJ26*, a t-DNA insertion line was available, and results based on this and rescue lines provided sufficient results. To clarify this, we corrected the subsection titles.

12. In section "The power and complexity of conditional GWAS", the authors write "The performance of GWAS relies on using the right model for the relation between genotype and phenotype. As with other statistical methods, using the wrong model may lead to unpredictable results." This seems like a too obvious of a statement.

Indeed: it is meant ironically. It is obvious, yet people do it.
